# The Genus *Echinops*: Phytochemistry and Biological Activities: A Review

**DOI:** 10.3389/fphar.2019.01234

**Published:** 2019-11-01

**Authors:** Helen Bitew, Ariaya Hymete

**Affiliations:** ^1^Department of Pharmacognosy, School of Pharmacy, College of Health Sciences, Mekelle University, Mekelle, Ethiopia; ^2^Department of Pharmaceutical Chemistry and Pharmacognosy, School of Pharmacy, College of Health Sciences, Addis Ababa University, Addis Ababa, Ethiopia

**Keywords:** *Echinops*, thiophene, phytochemistry, Asteraceae, pharmacological activity, traditional use

## Abstract

The genus *Echinops* belongs to the family of Asteraceae and comprises about 130 species. Many species belonging to the genus *Echinops* are traditionally used as medicinals mainly in Africa and Asia. The genus is reported to contain diverse secondary metabolites. The aim of this review is to critically evaluate the available research reports on the genus and systematically organize the findings. Information for this study was obtained using various search engines including PubMed and Google Scholar. This review revealed that the genus is used traditionally to treat pain, inflammation, respiratory diseases, diseases caused by different microorganisms, as an aphrodisiac, to fasten expulsion of placenta, and for removal of renal stones. More than 151 secondary metabolites have been reported from the genus in which thiophenic compounds held the biggest share. Various extracts, essential oils, and isolated compounds from members of this genus are shown to exhibit different biological effects mainly anti-microbial, anti-proliferative, and anti-inflammatory. However, there are a number of species in this genus that are claimed to have traditional medicinal uses but their biological effect not yet been evaluated.

## Introduction

*Echinops* L., belongs to the family of Asteraceae, a family which is distributed all over the world except in Antarctica. Asteraceae is a monophyletic taxon distinguished by florets arranged on a receptacle in centripetal heads and bounded by bracts. It comprises 1,600−1,700 genera and 24,000−30,000 species (Funk et al., 2005). The genus *Echinops* belongs to the tribe Cardueae and is recognized by the presence of uniflowered capitula aggregated into second-order spherical or oval heads. This feature makes it unique within the tribe (Garnatje et al., 2005; Sánchez-Jiménez et al., 2010). It contains 120−130 species distributed across north and tropical Africa, the Mediterranean Basin, and central Asia. Members of this genus are mostly perennial with few annuals (Hedberg et al., 2004; Sánchez-Jiménez et al., 2010).

Many members of this genus are traditionally used to treat different diseases. Some are scientifically investigated for various biological activities and phytoconstituents. Previously, reviews that focus on single species, *Echinops spinosus* L. and *E. echinatus* Roxb. have been conducted (Bouzabata et al., 2018; Maurya et al., 2015). To the authors' knowledge, there is no study that reviewed the traditional use, phytochemistry, and biological activities of the whole genus. This review is aimed to critically evaluate available research reports on the genus and systematically organize and present the findings. It is attempted to include all articles published from 1990−2018 while some articles published before 1990 were included considering their significance. This review excluded unpublished findings and publications which were not available online and articles written in languages other than English. Chemical structures of only isolated and characterized compounds were provided while structures of compounds identified from essential oils and other chemical analysis were not. The main sources of the structures of isolated compounds were the research articles and these were confirmed using PubChem. Structures that were not available in the articles were obtained from theses, books, PubChem, and other reliable sources. Different search engines including PubMed and Google Scholar were employed to search literature using searching words such as *Echinops*, plant, phytochemical, phytochemistry, pharmacological activity, biological effect, and traditional use.

## Traditional Uses

Ethnomedicinal claims on the genus *Echinops* to treat a number of ailments are depicted in **Table 1**. The common traditional uses can fall into three general groups. The frequently described application is to treat symptoms like inflammation, pain, and fever (Regassa, 2013; Rathore et al., 2015). The other common traditional use was to treat ailments related to respiratory tract including cough and sore throat (Ghasemi Pirbalouti et al., 2013; Sajjad et al., 2017). Members of the genus have been used as an aphrodisiac (Hamayun et al., 2006), facilitation of expulsion of retained placenta and delivery (Okello and Ssegawa, 2007; Qureshi and Bhatti, 2008), as an abortifacient (Abouri et al., 2012), treatment of uterus tumor (Abderrahim et al., 2013), and leucorrhoea (Wagh and Jain, 2018). Three species (*E. bannaticus* Rochel ex Schrad*, E. cornigerus* D.C., and *E. polyceras* Boiss.) reported to have been employed in the managment of kidney stones (Mustafa et al., 2012; Nawash et al., 2013; Kumar et al., 2018).

**Table 1:** Traditional uses of members of the genus *Echinops*.

Species	Part used	Indication	Country	Ref.
*E. amplexicaulis* Oliv.	R	HIV/AIDS	Uganda	Lamorde et al., 2010
	R	Ulcerative lymphagitis (LS)	Ethiopia	Fenetahun and Eshetu, 2017
		Stomachache	Ethiopia	Regassa et al., 2017
	R	Trypanosomiasis, liver disease, pasteurellosis	Ethiopia	Kitata et al., 2017
	R	Hydrocele	Uganda	Kamatenesi et al., 2011
	R	Fasten expulsion of placenta, hernia	Uganda	Okello and Ssegawa, 2007
	R	Ulcerative lymphagitis (LS)	Ethiopia	Tekle, 2014
*E. bannaticus* Rochel ex Schrad	R	Kidney stones	Kosovo	Mustafa et al., 2012
*E. bovei* (Boiss.) Maire.	AP	Eye complaints, trachoma, sores, inflammation, digestive diseases	Central Sahara	Hammiche and Maiza, 2006
*E. cornigerus* D.C.	R	Urinary problems mainly caused by kidney stones	India	Kumar et al., 2018
	WP	Insanity	India	Tiwari et al., 2010
	R	Removal of kidney stones	Pakistan	Jabeen et al., 2015
	R	Urinary complaints, fever	India	Dangwal et al., 2011
	WP	Cough, emergence of teeth in infants, fever, urinary trouble, tonic, sepsis, food poisoning	India	Sharma et al., 2012
	R	Urinary disorder, fever	India	Kumar and Pandey, 2015
	VP	Diuretic, aphrodisiac, fever, pain, chronic fever	Pakistan	Hamayun et al., 2006
	R	Fever, emergence of teeth in infants	India	Rathore et al., 2015
*E. echinatus* Roxb.	R	To treat hernia	India	Shende et al., 2018
	L	Earache	India	Maru et al., 2018
	R	Leucorrhoea	India	Wagh and Jain, 2018
	R, L	Joint pain	Pakistan	Malik et al., 2018
		Aphrodisiac, to facilitate the delivery process, abortifacient, leucorrhea, diabetes, eczema, heatstroke, wounds of cattle for killing maggots, liver disorders, cough, malarial fever, renal colic, lice, polyuria, appetite stimulant		Maurya et al., 2015
*E. giganteus* A. Rich	R	Anti-hemorrhoidal	Ethiopia	Desta, 1995
	R	Flatulence and bloody stool	Cameroon	Tacham et al., 2015
		Stomache, asthma attacks, as carminative	Cameroon	Menut et al., 1997
*E. hispidus* Fresen.	R and S	Sunstroke	Ethiopia	Meragiaw et al., 2016
*E. hoehnelii* Schweinf.	R	Internal parasite, amoebae, common cold	Ethiopia	Tekle, 2014
	R	Malaria, snakebite	Ethiopia	Giday et al., 2010
*E. kebericho* Mesfin	R	Black leg, respiratory manifestations, liver disease (LS)	Ethiopia	Yigezu et al., 2014
	Bl	Cough, headache	Ethiopia	Mesfin et al., 2014
	R	Scabies	Ethiopia	Amsalu et al., 2018
	R	Toothache, stomachache, common cold, sunstroke, tonsillitis, acute sickness, snake bite	Ethiopia	Regassa, 2013
	S	Fever, headache	Ethiopia	Gari et al., 2015
	R	Malaria, common cold	Ethiopia	Mekuanent et al., 2015
	R	Dislocated bone (LS)	Ethiopia	Teklay et al., 2013
	R	Toothache, vomiting, headache	Ethiopia	Abera, 2014
	R	Trypanosmiasis	Ethiopia	Shilema et al., 2013
	R	Gonorrhea	Ethiopia	Bizuayehu and Garedew, 2018
*E. longifolius* A. Rich.	RB	Headache, rheumatism, dry cough	Ethiopia	Suleman and Alemu, 2012
	R	Scorpion sting	Sudan	Issa et al., 2018
*E. macrochaetus* Fresen.	R	Toothache	Ethiopia	Belayneh and Bussa, 2014
	R	Headache	Ethiopia	Moravec et al., 2014
	Sd	Abdominal colic	Ethiopia	Gabriel and Guji, 2014
*E. niveus* Wall.	R	Diuretic, nerve tonic, cough, indigestion, ophthalmia. Applied to wounds in cattle to destroy maggots	India	Sharma et al., 2004
*E. polyceras* Boiss.	R	Kidney stones	Jordan	Nawash et al., 2013
*E. ritrodes* L.	S	Chronic cough	Urmia	Asadbeigi et al., 2014
	WP	Skin diseases, prevention of cough	Iran	Farouji and Khodayari, 2016
*E. sphaerocephalus* L.	R, S, L	Typhoid	Kenya	Nyang'au et al., 2017
*E. spinosissimus* Turra.	WP	Splenic diseases, sore throat	Saudi Arabia	El-Ghazali et al., 2010
	WP	Nerve tonic, diuretic, cough suppressant	UAE	Sajjad et al., 2017
	WP	Diuretic, nerve tonic, cough suppressant	Egypt	Mahmoud and Gairola, 2013
*E. spinosissimus* subsp. fontqueri (Pau) Greuter	R	Rheumatism, colds, uterus pains, uterus tumor	Morocco	Abderrahim et al., 2013
*E. spinosissimus* subsp. macro-plepis (Boiss.) Greuter	S,R, L	Renal disorders	Lebanon	Baydoun et al., 2015
*E. spinosus* L.	R	As hypoglycaemic, decoction is drunk.	Morocco	Merzouki et al., 2000
	R	Appetite stimulant, cold, diabetes, renal stones	Morocco	El Abbouyi et al., 2014
	L, S, R	Hepatoprotective, abortifacient	Morocco	Akdime et al., 2015
	R	Diabetes	Morocco	Katiri et al., 2017
	FAP	Colds, kidney stones, diuretic, hypoglycemic	Morocco	Abouri et al., 2012
	Br, R	Abortifacient, labor pain	Morocco	Abouri et al., 2012
	F	Neuralgia, tiredness	Morocco	Abouri et al., 2012
*E. spinosus* L. subsp Bovei (Boiss). Maire	R-Fr	Labor pains, abortifacient, neuralgia	Algeria	Chermat and Gharzouli, 2015
*E. viscidulus* Mozaff.	Bl	Cough, cold, sore throat	Iran	Ghasemi Pirbalouti et al., 2013
*E. viscosus* DC.	C	Boil	Turkey	Bulut et al., 2017

AP, Aerial part; B, Bark; Bl, Bulb; Br, Branch; C, Capitulum; F, Flower; FAP, Flowered aerial part; Fr, Fruit, L, Leaf; LS, Livestock; R, Root; RB, Root bark; S, Stem; Sd, Seed; VP, Vegetative part; WP, Whole plant.

In addition to the traditional medicnal applications described in **Table 1**, the plants have nutritional value. In Iran, the bulb of *E. viscidulus* Mozaff is consumed as a vegetable (Ghasemi Pirbalouti et al., 2013). The roots of *E. giganteus* A. Rich. and *E. spinosus* are used as a spice in Morocco and Cameroon, respectively (Pavela et al., 2016; Tbatou et al., 2016). The use of *E. giganteus* might be attributed to the presence of nutrients including iron, phenols, carotenoids, and vitamins E and C in the plant (Abdou Bouba et al., 2012).

## Phytochemicals

**Figure 1:**
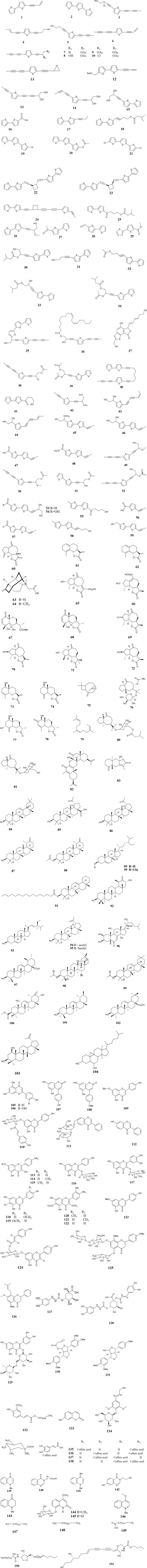
Structural formulae of secondary metabolites isolated from members of the genus *Echinops*.

As presented in **Table 2** and **Figure 1**, 151 compounds have been isolated and characterized using different spectroscopic/spectrometric techniques. Members of the genus *Echinops* contain primarily thiophenes and terpenes. Flavonoids and other phenolic compounds, alkaloids, lipids, and phenylpropanoids were also reported. The root of the plant is the main source of the thiophenes while most of the terpenes and flavonoids were isolated from the aerial part/the whole plant. The genus is also known for essential oil content and all morphological parts of the plants are reported to contain some of the essential oils. Around 53 of the isolated and characterized compounds are reported to have different biological activities. The structural formulae of isolated and characterized compounds are given in **Figure 1**.

**Table 2:** Secondary metabolites isolated from members of the genus *Echinops*.

No.	Name of secondary metabolites	Species	Plant part	Pharmacological activity	Ref.
Thiophenes					
**1.**	5-(but-3-en-1-ynyl)-2,2'-bithiophene	*E. macrochaetus*	R		Abegaz et al., 1991
		*E. pappii* Chiov.	R		Abegaz et al., 1991; Abegaz, 1991
		*E*. *ritro*	Rd	Antifungal	Fokialakis et al., 2006a
		*E. ritro*	AP		
		*E. latifolius*	R		Wang et al., 2006
		*E. grijsii*	R		Zhang and Ma, 2010; Chang et al., 2015
		*E. grijsii*	R	Cytotoxic	Jin et al., 2008
		*E. grijsii*	R	Insecticidal	Zhao et al., 2017
		*E. nanus* Bunge	R		Nakano et al., 2012
		*E. albicaulis*	AP		Kiyekbayeva et al., 2017
		*E. albicaulis*	WP	Termicidal	Fokialakis et al., 2006b
		*E. spinosissimus subsp. spinosissimus*	WP	Termicidal	Fokialakis et al., 2006b
**2.**	α-terthiophene	*E. ellenbeckii*	R		Abegaz et al., 1991
		*E. pappii*	R		Abegaz et al., 1991
		*E. macrochaetus*	R		
		*E. grijsii*	R	Cytotoxic	Jin et al., 2008
		*E. grijsii*	R		Liu et al., 2002; Zhang and Ma, 2010; Chang et al., 2015
		*E. grijsii*	R	Insecticidal	Zhao et al., 2017
		*E. latifolius*	R		Wang et al., 2006
		*E*. *ritro*	Rd	Antifungal	Fokialakis et al., 2006a
		*E. ritro*	AP	Termicidal	Fokialakis et al., 2006b
		*E. nanus*	R		Nakano et al., 2012
		*E. albicaulis*	R		Kiyekbayeva et al., 2017
		*E. albicaulis*	WP	Termicidal	Fokialakis et al., 2006b
		*E. albicaulis*	WP	Termicidal	Fokialakis et al., 2006b
		*E. albicaulis*	WP	Termicidal	Fokialakis et al., 2006b
		*E. transiliensis*	R	Insecticidal	Nakano et al., 2014
**3.**	5-(penta-1,3-diynyl)-2-(3-chloro-4-hydoxy-but-1-ynyl)-thiophene	*E. ellenbeckii*	R		Abegaz et al., 1991
		*E. giganteus*	R		
		*E. hispidus* Fresen.	R		
		*E. longisetus*	R		
		*E. macrochaetus*	R		
**4.**	Cis or trans-2-(pent-3-en-1-ynyl)-5-(4-hydroxybut-1-ynyl)-thiophenes	*E. pappii*	R		Abegaz, 1991
**5.**	5-(4-hydroxybut-1-ynyl)-2-(pent-1,3-diynyl)-thiophene	*E. pappii*	R		Abegaz, 1991
		*E*. *ritro*	Rd	Antifungal	Fokialakis et al., 2006a
		*E. ritro*	AP	Termicidal	Fokialakis et al., 2006b
		*E. grijsii*	R		Chang et al., 2015
		*E. grijsii*	R	Cytotoxic	Zhang et al., 2009;
		*E. grijsii*	R	NQO1-inducing	Zhang and Ma, 2010
		*E. giganteus*	Rz	Cytotoxic	Kuete et al., 2013
		*E. giganteus*	Rz	Cytotoxic	Sandjo et al., 2016
**6.**	5-(penta-1,3-diynyl)-2-(but-3-en-1-ynyl)-thiophene	*E. ellenbeckii*	R		Hymete et al., 2005b
**7.**	5-(penta-1,3-diynyl)-2-(4-acetoxy-but-1-ynyl)-thiophene	*E. ellenbeckii*	R		Hymete et al., 2005b
**8.**	5-(penta-1,3-diynyl)-2-(3-hydroxy-4-acetoxy-but-1-ynyl)-thiophene	*E. ellenbeckii*	R		Hymete et al., 2005b
		*E*. *hoehnelii*	R		Bitew et al., 2017
		*E. transiliensis*	R	Insecticidal	Nakano et al., 2014
**9.**	5-(penta-1,3-diynyl)-2-(3,4-diacetoxy-but-1-ynyl)-thiophene	*E. ellenbeckii*	R		Hymete et al., 2005a
		*E. grijsii*	R		Jin et al., 2008
		*E. grijsii*	R	NQO1-inducing	Zhang and Ma, 2010
		*E. transiliensis*	R	Insecticidal	Nakano et al., 2014
**10.**	5-(penta-1,3-diynyl)-2-(3-chloro-4-acetoxy-but-1-ynyl)-thiophene	*E. ellenbeckii*	R		Hymete et al., 2005b
		*E. transiliensis*	R	Insecticidal	Nakano et al., 2014
		*E. albicaulis*	WP	Termicidal	Fokialakis et al., 2006b
		*E. hoehnelii*	R	Anti-malarial	Bitew et al., 2017
**11.**	5-(penta-1,3-diynyl)-2-(3,4-epoxy-but-1-ynyl)-thiophene	*E. ellenbeckii*	R		Hymete et al., 2005b
**12.**	5-[-(5-acetoxymethyl-2-trienyl)-2-(but-3-ene-1-ynyl)]-thiophene	*E. ellenbeckii*	R		Hymete et al., 2005b
**13.**	5-(5,6-dihydroxy-hexa-1,3-diynyl)-2-(prop-1-ynyl)-thiophene (echinoynethiophene A)	*E. grijsii*	R		Liu et al., 2002; Dong et al., 2008a
		*E. grijsii*	R	Cytotoxic	Zhang et al., 2009
**14.**	5-(penta-1,3-diynyl)-2-(3,4-dihydroxybut-1-ynyl)-thiophene	*E. grijsii*	R		Liu et al., 2002; Dong et al., 2008a; Chang et al., 2015
		*E. ritro*	WP		Li et al., 2019
		*E. grijsii*	R	NQO1-inducing	Shi et al., 2010; Zhang and Ma, 2010
		*E. grijsii*	R	Cytotoxic	Zhang et al., 2009
		*E. giganteus*	Rz	Cytotoxic	Sandjo et al., 2016
		*E. transiliensis*	R	Insecticidal	Nakano et al., 2014
		*E*. *hoehnelii*	R	Anti-malarial	Bitew et al., 2017
**15.**	5-(3,4-dihydroxybut-1-ynyl)-2,2′-bithiophene	*E. grijsii*	R		Liu et al., 2002; Dong et al., 2008a; Zhang et al., 2009; Zhang and Ma, 2010; Chang et al., 2015
		*E. ritro*	WP		Li et al., 2019
		*E. latifolius*	R	Cytotoxic	Wang et al., 2007
		*E. transiliensis*	R	Insecticidal	Nakano et al., 2014
**16.**	2,2'-bithiophene-5-carboxylic acid	*E. grijsii*	R		Liu et al., 2002; Chang et al., 2015
		*E. ritro*	WP		Li et al., 2019
**17.**	5-(3-buten-1-ynyl)-2,2'-bithiophene	*E. grijsii*	R		Liu et al., 2002
**18.**	5-(4-isovaleroyloxybut-1-ynyl)-2,2'-bithiophene	*E. grijsii*	R		Liu et al., 2002; Chang et al., 2015
		*E. grijsii*	R		Wang et al., 2006
		*E. grijsii*	R	Cytotoxic	Jin et al., 2008
		*E. grijsii*	R	Insecticidal	Zhao et al., 2017
		*E*. *ritro*	Rd	Antifungal	Fokialakis et al., 2006a
		*E. ritro*	AP	Termicidal	Fokialakis et al., 2006b
**19.**	5-chloro- α-terthiophene	*E. grijsii*	R		Liu et al., 2002
**20.**	5-acetyl α-terthiophene	*E. grijsii*	R		Liu et al., 2002
**21.**	5,5'-dichloro-α-terthiophene	*E. grijsii*	R		Liu et al., 2002
**22.**	Cardopatine	*E. grijsii*	R		Liu et al., 2002; Chang et al., 2015
		*E. latifolius*	R		Wang et al., 2006
		*E*. *ritro*	Rd	Antifungal	Fokialakis et al., 2006a
		*E. ritro*	AP	Termicidal	Fokialakis et al., 2006b
**23.**	Isocardopatine	*E*. *grijsii*	R		Liu et al., 2002; Zhang and Ma, 2010; Chang et al., 2015
		*E*. *ritro*	Rd	Antifungal	Fokialakis et al., 2006a
		*E. grijsii*	R	Cytotoxic	Jin et al., 2008
**24.**	Grijisyne A	*E. grijsii*	R		Zhang et al., 2008
**25.**	Grijisone A	*E. grijsii*	R		Zhang et al., 2008
**26.**	5-(4-hydroxy-3-methoxy-1-butyny)-2,2'-bithiophene	*E. grijsii*	R		Chang et al., 2015
**27.**	5-acetyl-2,2'-bithiophene	*E. latifolius*	R		Wang et al., 2008
		*E. grijsii*	R		Chang et al., 2015
		*E. ritro*	WP		Li et al., 2019
**28.**	5-formyl-2,2'-bithiophene	*E. grijsii*	R		Chang et al., 2015
**29.**	Methyl 2,2'-bithiophene-5-carboxylate	*E. grijsii*	R		Chang et al., 2015
**30.**	5-(3-hydroxymethyl-3-isovaleroyloxyprop-1-ynyl)-2,2'-bithiophene	*E. latifolius*	R		Wang et al., 2006
		*E. grijsii*	R		Chang et al., 2015
**31.**	5-(4-hydroxy-1-butynyl)-2,2'-bithiophene	*E. latifolius*	R		Wang et al., 2008
		*E. ritro*	Rd	Antifungal	Fokialakis et al., 2006a
		*E. latifolius*	R	Cytotoxic	Wang et al., 2007
		*E. grijsii*	R		Zhang et al., 2009; Chang et al., 2015
		*E. ritro*	WP	Antibacterial, Antifungal	Li et al., 2019
		*E. ritro*	AP	Termicidal	Fokialakis et al., 2006b
**32.**	5-(4-acetoxy-1-butynl)-2,2'-bithiophene	*E. grijsii*	R		Chang et al., 2015
**33.**	5-(3-hydroxy-4-isovaleroyloxybut-1-ynyl)-2,2'-bithiophene	*E. latifolius*	R		Wang et al., 2006
**34.**	5-(3-acetoxy-4-isovaleroyloxybut-1-ynyl)-2,2′-bithiophene	*E. latifolius*	R		Wang et al., 2006
		*E. grijsii*	R	Cytotoxic	Jin et al., 2008
**35.**	Echinopsacetylenes A	*E. transiliensis*	R		Nakano et al., 2011
**36.**	Echinopsacetylenes B	*E. transiliensis*	R		Nakano et al., 2011
**37.**	Echinothiophenegenol	*E. grijsii*	R		Zhang et al., 2009
		*E. nanus*	R		Nakano et al., 2012
**38.**	5-(4-acetoxy-3-chlorobut-1-ynyl)-2-(pent-1,3-diynyl)-thiophene	*E*. *ritro*	Rd	Antifungal	Fokialakis et al., 2006a
**39.**	5-(3,4-diacetoxybut-1-ynyl)-2,2′-bithiophene	*E*. *ritro*	Rd	Antifungal	Fokialakis et al., 2006a
		*E. ritro*	AP	Termicidal	Fokialakis et al., 2006b
		*E. grijsii*	R		Zhang and Ma, 2010
		*E. grijsii*	R	Cytotoxic	Jin et al., 2008
		*E. transiliensis*	R	Insecticidal	Nakano et al., 2014
**40.**	5-{4-[4-(5-pent-1,3-diynylthiophene-2-yl)-but-3-yny}-2,2'-bithiophene	*E. latifolius*	R	Cytotoxic	Wang et al., 2007
**41.**	5-(4-hydroxybut-1-one)-2,2'-bithiophene	*E. latifolius*	R	Cytotoxic	Wang et al., 2007
		*E. ritro*	WP		Li et al., 2019
**42.**	5-(prop-1-ynyl)- 2-(3,4-diacetoxybut-1-ynyl)-thiophene	*E. latifolius*	R		Wang et al., 2007
		*E. grijsii*	R	Cytotoxic	Jin et al., 2008
**43.**	5-(1,2-dihydroxy-ethyl)-2-(Z)-hept-5-ene-1,3-diynylthiophene	*E. latifolius*	R	Anti-inflammatory	Jin et al., 2016
**44.**	5-(1,2-dihydroxyethyl)-2-(*E*)-hept-5-ene-1,3-diynylthiophene	*E. latifolius*	R	Anti-inflammatory	Jin et al., 2016
**45.**	6-Methoxy-arctinol-b	*E. latifolius*	R	Anti-inflammatory	Jin et al., 2016
**46.**	Arctinol-b	*E. grijsii*	R		Zhang et al., 2009
		*E. latifolius*	R	Anti-inflammatory	Jin et al., 2016
		*E. ritro*	WP	Antibacterial, Antifungal	Li et al., 2019
**47.**	Arctinol	*E. latifolius*	R	Anti-inflammatory	Jin et al., 2016
		*E. ritro*	WP		Li et al., 2019
		*E. ritro*	WP		Li et al., 2019
**48.**	Methyl [5'-(1-propynyf)-2,2'-bithienyl-5-yl] carboxylate	*E. latifolius*	R	Anti-inflammatory	Jin et al., 2016
**49.**	5-(penta-1,3-diynyl)-2-(3-methoxy-4-hydroxy-but-1-ynyl)-thiophene	*E*. *hoehnelii*	R		Bitew et al., 2017
**50.**	5-(penta-1,3-diynyl)-2-(3-methoxy-4-acetoxy-but-1-ynyl)-thiophene	*E*. *hoehnelii*	R		Bitew et al., 2017
**51.**	5-(3-hydroxy-4-acetoxybut-1-ynyl)-2,2′-bithiophene	*E. transiliensis*	R		Nakano et al., 2014
		*E. transiliensis*	R	Insecticidal	Nakano et al., 2014
**52.**	5-(penta-1,3-diynyl)-2-(3-acetoxy-4-hydroxy-but-1-ynyl)-thiophene	*E. transiliensis*	R	Insecticidal	Nakano et al., 2014
**53.**	5'-(3,4-dihydroxybut-1-yn-1-yl)-[2,2'-bithiophene]-5-carbaldehyde.	*E. ritro*	WP		Li et al., 2019
**54.**	5'-(3,4-dihydroxybut-1-yn-1-yl)-[2,2'-bithiophene]-5-carboxylic acid	*E. ritro*	WP	Antibacterial	Li et al., 2019
**55.**	4-hydroxy-1-(5'-methyl-[2,2'-bithiophen]-5-yl)butan-1-one	*E. ritro*	WP	Antibacterial, Antifungal	Li et al., 2019
**56.**	Junipic acid	*E. ritro*	WP		Li et al., 2019
**57.**	Arctinal	*E. ritro*	WP	Antibacterial	Li et al., 2019
**58.**	4-(5'-methyl-[2,2'-bithiophen]-5-yl)but-3-yn-1-ol	*E. ritro*	WP		Li et al., 2019
**59.**	Arctinol A	*E. ritro*	WP	Antibacterial	Li et al., 2019
**Terpenes**					
**60.**	Dehydrocostus lactone	*E. amplexicauli*	R		Abegaz et al., 1991
		*E. kebericho*			
**61.**	Costunolide	*E. amplexicaulis*,			[Bibr B8][Bibr B7]
		*E. kebericho*,			
		*E. pappii*			
**62.**	Dihydrocostunolide	*E. amplexicaulis*	R		Abegaz et al., 1991
**63.**	Echinopines A	*E. spinosus*	R		Dong et al., 2008b
**64.**	Echinopines B	*E. spinosus*	R		Dong et al., 2008b
**Terpenes**					
**65.**	(3α,4α,6α)-3,13-dihydroxyguaia-7(11),10(14)-dieno-12,6-lactone)	*E. ritro*	WP		Li et al., 2010
**66.**	(3α,4α,6α,11ß)-3-hydroxyguai-1(10)-eno-12,6-lactone)	*E. ritro*	WP		Li et al., 2010
**67.**	(11α)-11,13-dihydroarglanilic acid methyl ester	*E. ritro*	WP		Li et al., 2010
**68.**	Vulgarin	*E. ritro*	WP		Li et al., 2010
**69.**	(3*R*,3a*S*,6a*R*,9*S*,9a*R*,9b*S*)-octahydro-3,9-dimethyl-6-methyleneazuleno[4,5-b]furan2,8(3*H*,9b*H)-*d ione	*E. ritro*	WP		Li et al., 2010
**70.**	(3a*S*,6a*R*,8*S*,9*S*,9a*R*,9b*R*)-decahydro-8-hydroxy-9-methyl-3,6 dimethyleneazuleno[4,5-b]furan-2(9b*H*)-one	*E. ritro*	WP		Li et al., 2010
**71.**	(3a*S*,6a*R*,8*R*,9*R*,9a*R*,9b*R*)-decahydro-8-hydroxy-3,3,9-trimethyl-6-methyleneazuleno[4,5-b]furan-2(9b*H*)-one	*E. ritro*	WP		Li et al., 2010
**72.**	(3*R*,3a*S*,6a*R*,8*S*,9*S*,9a*R*,9b*S*)-decahydro-8-hydroxy-3,9-dimethyl-6-methyleneazuleno[4,5-b]furan-2(9b*H*)-one	*E. ritro*	WP		Li et al., 2010
**73.**	Santamarin	*E. pappii*			Abegaz, 1991
		*E. ritro*	WP		Li et al., 2010
**74.**	Reynosin	*E. pappii*	R		Abegaz, 1991
**75.**	Caryophyllene epoxide	*E. giganteus*	R		Abegaz et al., 1991
		*E. hispidus*			
**76.**	Echusoside	*E. hussoni* Boiss.	AP		Ka, 2001
**77.**	(3*S*,3a*S*,5a*R*,6*R*,8*R*,9b*S*)-decahydro-6,8-dihydroxy-3,5a-dimethyl-9-methylenenaphtho[1,2-b]furan-2(*9bH*)-one	*E. ritro*	WP		Li et al., 2010
**78.**	(3*S*,3a*S*,5a*R*,6*S*,9b*S*)-3,3a,4,5,5a,6-hexahydro-6-hydroxy-3,5a,9-trimethylnaphtho[1,2-b]furan-2,7(9a*H*,9b*H*)-dione	*E. ritro*	WP		Li et al., 2010
**79.**	2,6,10-trimethyldodeca-2,6,10-triene	*E. albicaulis*	AP		Kiyekbayeva et al., 2017
**80.**	Macrochaetosides A	*E. macrochaetus*	AP	Cytotoxic	Zamzami et al., 2019
**81.**	Macrochaetosides B	*E. macrochaetus*	AP	Cytotoxic	Zamzami et al., 2019
**82.**	Latifolanone A	*E. latifolius*	R		Jin et al., 2016
**83.**	Atractylenolide-II	*E. latifolius*	R	Anti-inflammatory	Jin et al., 2016
**84.**	ß-amyrin	*E. niveus*	WP		Singh et al., 1990
**85.**	Betulinic acid	*E. niveus*	WP		
**86.**	Lupeol	*E. niveus*	WP		Singh et al., 1990
		*E. giganteus*	R		Tene et al., 2004
		*E. integrifolius*	WP		Senejoux et al., 2013
		*E. echinatus*	R		Patel, 2016
**87.**	Taraxasterol	*E. niveus*	WP		Singh et al., 1990
**88.**	Taraxasterol acetate	*E. niveus*	WP		Singh et al., 1990
		*E. echinatus*	WP	Anti-inflammatory	Singh et al., 1989
**89.**	ß-sitosterol	*E. niveus*	WP		Singh et al., 1990
		*E. transiliensis*	R		Nakano et al., 2012
		*E. giganteus*	Rz		Kuete et al., 2013
		*E. orientalis*	Sd	Antioxidant	Erenler et al., 2014
**90.**	ß-sitosterol glucoside	*E. niveus*	WP		Singh et al., 1990
		*E. giganteus*	R		Tene et al., 2004
		*E. integrifolius*	WP		Senejoux et al., 2013
		*E. albicaulis*	AP		Kiyekbayeva et al., 2017
**91.**	Reynosin	*E. pappii*	R		Abegaz, 1991
**92.**	Gmeliniin A	*E. gmelinii*	AP		He et al., 2000
**93.**	Stigmasterol	*E. transiliensis*	R		Nakano et al., 2012
		*E. macrochaetus*	AP		Zamzami et al., 2019
		*E. integrifolius*	WP		Senejoux et al., 2013
		*E. giganteus*	Rz		Kuete et al., 2013
**94.**	Lupeol acetate	*E. integrifolius*	WP		Senejoux et al., 2013
		*E. echinatus*	R		Patel, 2016
		*E. albicaulis*	AP		Kiyekbayeva et al., 2017
**95.**	Lupeol linoleate	*E. albicaulis*	AP		Kiyekbayeva et al., 2017
**96.**	Ajugasterone C	*E. grijisii*	R		Dong et al., 2008a
**97.**	Ursolic acid	*E. giganteus*	Rz	Cytotoxic	Kuete et al., 2013
**98.**	Echinopsolide A (3ß-acetoxy-15α-bromoolean-13ß,28-olide)	*E. giganteus*	Rz	Cytotoxic	Sandjo et al., 2016
**99.**	ß-amyrin acetate	*E. giganteus*	Rz		Sandjo et al., 2016
**100.**	3ß-acetoxy-taraxast-12,20(30)-diene-11α-21α-diol	*E. galalensis*	AP	Hepato-protective	Abdallah et al., 2013
**101.**	α-amyrin	*E. galalensis*	Rz	Hepato-protective	
**102.**	Erythrodiol	*E. galalensis*	Rz	Hepato-protective	
**Terpenes**					
**103.**	Lup-20(29)-ene-1,3-diol	*E. galalensis*	Rz	Hepato-protective	
**104.**	Cyclostenol	*E. macrochaetus*	AP	Cytotoxic	Zamzami et al., 2019
**Flavonoids and other phenolic compounds**					
**105.**	Apigenin	*E. niveus*	WP		Singh et al., 1990
		*E. echinatus*			Ram et al., 1995
		*E. integrifolius*	AP		Senejoux et al., 2013
		*E. spinosus*	AP		Boumaraf et al., 2016
		*E. albicaulis*	AP		Kiyekbayeva et al., 2017
**106.**	Luteolin	*E. niveus*	WP		Singh et al., 1990
		*E. grijisii*	R		Dong et al., 2008a
**107.**	Nivegin	*E. niveus*	WP		Singh et al., 1990
**108.**	Nivetin	*E. niveus*	AP		Singh and Pandey, 1990
**109.**	Apigenin 7-*O*-glucoside	*E. echinatus*	F		Ram et al., 1995
		*E. spinosus*	AP		Boumaraf et al., 2016
		*E. orientalis*	Sd	Antioxidant	Erenler et al., 2014
**110.**	Echitin	*E. echinatus*	F		Ram et al., 1995
**111.**	Echinoside	*E. echinatus*	WP		Singh et al., 2006
**112.**	7-hydroxyisoflavone	*E. echinatus*	WP		Singh et al., 2006
**113.**	Kaempferol	*E. echinatus*	WP		Singh et al., 2006
**114.**	Kaempferol-4'-methylether	*E. echinatus*	WP		Singh et al., 2006
**115.**	Kaempferol-7-methylether	*E. echinatus*	WP		Singh et al., 2006
**116.**	Kaempferol-3-*O*-α-L-rhamnoside	*E. echinatus*	WP		Singh et al., 2006
		*E. heterophyllus*	AP		Mahmood and Khadeem, 2013
**117.**	Myrecetin-3-*O*-α-L-rhamnoside	*E. echinatus*	WP		Singh et al., 2006
**118.**	Chrysoeriol	*E. integrifolius*	WP		Senejoux et al., 2013
**119.**	Hispidulin	*E. integrifolius*	WP		Senejoux et al., 2013
**120.**	Jaceidin	*E. integrifolius*	WP		Senejoux et al., 2013
**121.**	Centaureidin	*E. integrifolius*	WP		Senejoux et al., 2013
**122.**	Axillarin	*E. integrifolius*	WP		Senejoux et al., 2013
**123.**	Genkwanin	*E. albicaulis*	AP		Kiyekbayeva et al., 2017
**124.**	Apigenin-7-*O*-(6"-trans-pcoumaroyl- ß -D-glucopyranoside	*E. orientalis*	L	Antioxidant	Erenler et al., 2014
		*E. spinosus*	AP		Boumaraf et al., 2016
**125.**	5,7-dihydroxy-8,4'-dimethoxyflavanone-5-*O*-α-L-rhamno-pyranosyl-7-*O*-ß-D-arabinopyranosyl (1→4)-*O*-ß-D-glucopyranoside	*E. echinatus*	WP	Anti-inflammatory	Yadava and Singh, 2006
**126.**	Candidone	*E. giganteus*	Rz	Cytotoxic	Kuete et al., 2013
**127.**	Chlorogenic acid	*E. grijisii*	R		Dong et al., 2008a
**128.**	Cynarin	*E. grijisii*	R		Dong et al., 2008a
**129.**	Rutin	*E. heterophyllus*	AP		Mahmood and Khadeem, 2013
		*E. albicaulis*	AP		Kiyekbayeva et al., 2017
**130.**	(+)-4-(3-methylbutanoyl)-2,6-di(3,4-dimethoxy)phenyl-3,7-dioxabicyclo[3.3.0]octane	*E. giganteus*	R		Tene et al., 2004
**131.**	(+)-4-hydroxy-2,6- di(3,4-dimethoxy)phenyl-3,7-dioxabicyclo[3.3.0]octane	*E. giganteus*	R		Tene et al., 2004
		*E. giganteus*	Rz		Sandjo et al., 2016
		*E. giganteus*	Rz	Cytotoxic	Kuete et al., 2013
**132.**	Hexacosyl-(*E*)-ferulate	*E. nanus*	R		Nakano et al., 2012
**133.**	Umbelliferone	*E*. *integrifolius*	WP		Senejoux et al., 2013
**134.**	Syringin	*E. grijisii*	R		Dong et al., 2008a
**135.**	1,5-dicaffeoylquinic acid	*E. galalensis*	AP	Hepato-protective	Abdallah et al., 2013
**136.**	3,5-dicaffeoylquinic acid			Hepato-protective	
**137.**	3,4-dicaffeoylquinic acid			Hepato-protective	
**138.**	4,5-dicaffeoylquinic acid			Hepato-protective	
**Alkaloids**					
**139.**	Echinopsine (1-methyl-4-quinolone)	*E. echinatus*	AP		Chaudhuri, 1987
		*E. nanus*	R		Nakano et al., 2012
		*E. albicaulis*	AP		Kiyekbayeva et al., 2017
		*E. orientalis*	Sd	Antioxidant	Erenler et al., 2014
**140.**	Echinozolinone	*E. echinatus*	AP		Chaudhuri, 1987
**Alkaloids**					
**141.**	Echinopsidine	*E. echinatus*	AP		Chaudhuri, 1987
**142.**	7-hydroxyechinozolinone	*E. echinatus*	F		Chaudhuri, 1992
**143.**	1-Methyl-4(*1H*)-quinolinone	*E. heterophyllus*	Sd		Khadim et al., 2014
**144.**	1-methyl-4-methoxy-8-(ß-D-glucopyranosyloxy)-2(*1H*)-quinolinone	*E. gmelinii Turcz.*	AP		Su et al., 2004
**145.**	4-methoxy-8-(-D-glucopyranosyloxy)-2(*1H*)-quinolinone	*E. gmelinii*	AP		Su et al., 2004
**146.**	Echinorine	*E. albicaulis*	AP		Kiyekbayeva et al., 2017
**Lipids**					
**147.**	Triacontane	*E*. *integrifolius*	R		Karimov and Aisa, 2013
**148.**	Heptacosane	*E*. *integrifolius*	R		Karimov and Aisa, 2013
**149.**	Lignoceric acid	*E*. *integrifolius*	R		Karimov and Aisa, 2013
**150.**	Tetrahydrofurano-ceramide	*E. giganteus*	Rz	Cytotoxic	Sandjo et al., 2016
**151.**	Ritroyne A	*E. ritro*	R		Li et al., 2019

AP, Aerial part; F, Flower; L, Leaf; R, Root; Rd, Radix; Rz, Rhizome; Sd, Seed; WP, Whole plant.

### Thiophenes

Thiophenes, the main bioactive constituents of the genus *Echinops*, are biosynthetically derived from fatty acids and reduced sulphur (Arroo et al., 1997). Majority of the thiophenic compounds comprise an acetylenic functional group and most of the thiophenes comprised two thiophene rings in their structure. The most abundant thiophenes which were reported from nine species were 5-(but-3-en-1-ynyl)-2,2'-bithiophene (**1**) and α-terthiophene (**2**). 5-(4-hydroxybut-1-ynyl)- 2-(pent-1,3-diynyl)-thiophene (**5**), 5-(penta-1,3-diynyl)-2-(3,4-dihydroxybut-1-ynyl)-thiophene (**14**), and 5-(4-hydroxy-1-butynyl)-2,2'-bithiophene (**31**) were isolated from five species. Thiophenes were detected in essential oils obtained from the different plants of this genus. 5-(3-buten-1-ynyl)-2,2'-bithienyl was detected in essential oils obtained from the roots of *E. grijsii* Hance, *E*. *bannaticus*, and *E*. *sphaerocephalus* L.

The biological activities of thiophenes were evaluated mainly *in vitro* and they have an insecticidal, anti-proliferative, and anti-fungal potential effects.

### Terpenoids

Sesqui- and triterpenoids were reported mainly from the whole plant and aerial parts of the genus *Echinops*. Most of the sesquiterpenoids contain lactones. Sesquiterpene lactones are also the most prevalent secondary metabolites in the family of Asteraceae (Chadwick et al., 2013). Most triterpenoids exist in various forms including lactones, esters, and sterols along with their glycosides. The common sesquiterpenoid reported was costunolide (**61)**, which was isolated from three species whereas lupeol (8**6**) and lupeol acetate (**94**) were the common triterpenoids. Many sesquiterpenoids were also detected from the essential oils of the genus.

### Flavonoids and Other Phenolic Compounds

Flavonoids from the genus *Echinops* were mainly flavones and mostly isolated from the whole plant and aerial parts of the members. Apigenin (**105**) is the most common flavonoidal aglycone and it was isolated from the flower and whole plant of *E. niveus* Wall., *E. echinatus, E. integrifolius* Kar. & Kir., and *E. albicaulis* Kar. & Kir. (**Table 2**). In addition to flavonoids, phenolic compounds including coumarins, phenylpropanoids, and lignans were reported (Tene et al., 2004; Dong et al., 2008a; Senejoux et al., 2013).

### Alkaloids

The first alkaloids isolated from the genus *Echinops* were echinopsine (**139**), echinozolinone (**140**), and echinopsidine (**141**) from the aerial parts of *E. echinatus* (Chaudhuri, 1987). Later on, another alkaloid, 7-hydroxyechinozolinone (**142**), was isolated from the flowers of the same plant (Chaudhuri, 1992). Additional four alkaloids of which two were in glycosidic form were reported (**Table 2**). The alkaloids were mainly isolated from the aerial parts of the plants. The predominant alkaloid, which was isolated from four different species, was 1-methyl-4-quinolone (139).

### Essential Oils and Lipids

The genus *Echinops* is rich in bioactive essential oil constituents, which were mainly found in the roots. Various reports indicated the presence of terpenoids and thiophenes.

The root of *E. grijisii* was found to contain *cis*-β-farnesene and 5-(3-buten-1-ynyl)-bithiophene as main components (Guo et al., 1994). Essential oils from root, stem, leaf, and flowers of *E. ellenbeckii* comprised mainly β-maaliene, cyperene, caryophyllene oxide, and β-selinene from the respective plant parts (Hymete et al., 2004). The fresh inflorescences of *E. graecus* and *E. ritro* yielded methyl chavicol and (*E*)-2-hexenal, 1,8-cineole, and *p*-cymene as major constituents, respectively (Papadopoulou et al., 2006).

Essential oils from the root of *E. bannaticus* and *E. sphaerocephalus* were reported to contain 5-(3-buten-1-ynyl)-2,2'-bithienyl and α-terthienyl as major constituents, and also triquinane sesquiterpenoids (Radulović and Denić, 2013). The most abundant compounds from *E. giganteus* have been reported to be tricyclic sesquiterpenoids such as silphiperfol-6-ene and presilphiperfolan-8-ol followed by presilphiperfol-7-ene, cameroonan-7-α-ol, and (*E*)-caryophyllene (Pavela et al., 2016).

Ceramides, sulf-polyacetylene ester, and simple hydrocarbons were the nonpolar constituents from the genus (**Figure 1**). The ethyl acetate extract of *E*. *integrifolius* contained lupeolacetate, 1,3-butadiene-1-carboxylic acid, lupeol, (1*R*,3*R*,4*R*,5*R*)-(–)-quinic acid, palmitic acid, and D-*threo*-*O*-ethylthreonine as the main constituents (Karimov and Aisa, 2012). In a related study, GS-MS analysis of petroleum ether extract of the aerial part of *E*. *integrifolius* indicated the presence of methyl esters of fatty acids as well as saturated hydrocarbons such as octacosane, hentriacontane, hexacosane, tetratetraacontane, eicosane, and nonadecane. Trace amount of 2-octanone and 4,8,12,16-tetramethyl heptadecan-4-olide were also detected in *E. integrifolius* (Karimov and Aisa, 2013).

## Biological Activities

### Anti-Microbial Activity

The genus *Echinops* is traditionally used to treat different infectious diseases including trachoma, sepsis, typhoid, gonorrhea, and ulcerative lymphangitis. It is also used to treat different ailments that might be caused by bacterial/fungal infections including fever, respiratory diseases, toothache, leucorrhoea, and earache. Thus, they have been investigated for their anti-microbial activities. Anti-bacterial and anti-fungal activities of extracts from the genus with their respective minimum inhibitory concentration (MIC), minimum bactericidal concentration (MBI), minimum fungicidal concentration (MFC), and zone of inhibitions are presented in **Table 3**. These studies showed that both Gram-positive and Gram-negative bacteria were sensitive to the extracts/isolated compounds obtained from the genus.

**Table 3:** *In vitro* antibacterial and antifungal activities of some *Echinops* species.

*Echinops* species	Extract(Plant part)	Strain (ID)	Type	MIC	MBC	Zone of inhibition (mm) (Conc.) (mg/mL)	Ref.
*E. adenocaulos* Bioss.	Zamzam water	*Streptococcus pneumonia* (MDR)	I	780 µg/mL	–	–	Saleh Fares et al., 2013
*E. amplexicaulis*	Ether (R)	*Mycobacterium tuberculosis* (MDR)	I	50 µg/mL	50 µg/mL	41.0 (50)	Kevin et al., 2018
		*M.tuberculosis* (H37Rv)	S	12 µg/mL	10 µg/mL	40.3 (50)	
		*M. bovis* (BCG strain)	S	45 µg/mL	50 µg/mL	40. 7 (50)	
*E. echinatus*	70% Ethanol (AP)	*Bacillus subtilis*	S			14.7 (0.8)	Ahmad, 2012
		*S. aureus*	S			12.7 (0.8)	
		*Pseudomonas aeruginosa*	S			20.7 (0.8)	
		*Salmonella typhi*	S			27.3 (0.8)	
		*Shigella sonnei*	S			17.3 (0.8)	
		*Escherichia coli*	S			24 (0.8)	
*E*. *ellenbeckii*	80% Methanol (L)	*S. aureus* (ATCC 6538)	S	–	–	23.0 (10)	Hymete et al., 2005a
*E*. *giganteus*	Methanol (R)	*Klebsiella pneumonia* (K24)	LC	32 µg/mL	–	–	Fankam et al., 2011
		*M. tuberculosis* (H37Rv)	CC	32 µg/mL	128 µg/mL		Tekwu et al., 2012
		*M. tuberculosis* (H37Ra)	CC	16 µg/mL	128 µg/mL		
*E. kebericho*	Water/Ethanol/Methanol (R)	*S. aureus*	S	100/3.1/3.1 µg/mL	> 100/6.3/9.4 µg/mL	8.3/19.3/18(0.08)	Ameya et al., 2016
	Ethanol/Methanol (R)	*E. fecalis*	S	12.5/12.5 µg/mL	18.75/18.75 µg/mL	11.66/14.1(0.08)	
		*E. coli*	S	25/25 µg/mL	37.5/37.5 µg/mL	9.66/8.66 (0.08)	
	Essential oils (R)	*Listeria monocytogenes*	S	0.2 µL/mL	0.4 µL/mL	–	Belay et al., 2011
		*S. aureus*		0.2 µL/mL	0.4 µL/mL		
		*S. pyogenes*		0.2 µL/mL	0.8 µL/mL		
		*P. aeruginosa*		0.2 µL/mL	0.4 µL/mL		
		*Shigella dysenteriae*		6.3 µL/mL	6.3 µL/mL		
		*K. pneumonia*		0.1 µL/mL	0.2 µL/mL		
		*Proteus mirabilis*		25 µL/mL	25 µL/mL		
		*Bacillu scereus*		0.4 µL/mL	0.8 µL/mL		
*E*. *longisetus*	80% Methanol (L)	*S. aureus* (ATCC 6538)	S	–	–	23.0 (10)	Hymete et al., 2005a
	80% Methanol (St)	*S. aureus* (ATCC 6538)	S	–	–	23.3 (10)	
*E. ritro*	Essential oil (AP)	*S. aureus* (ATCC 25923)	S	150 µg/mL			Jiang et al., 2017
		*Salmonella Enteritidis* (CICC21513)	S	600 µg/mL			
*E. spinosissimus*	Methanol (AP)	*B. cereus*	I	–	–	12 (6)	Rahman et al., 2011
	Methanol (AP)	*S. aureus*	I	–	–	12 (6)	
	Methanol (AP)	*S. aureus*	I	–	–	10 (6)	
	Methanol (AP)	*S. aureus*	I	–	–	32 (6)	
	Hexane (AP)	*S. sepidermis*	I	–	–	16 (6)	
	Methanol (AP)	*E. coli*	I	–	–	11 (6)	
	Methanol (AP)	*Klebsiella oxytoca*	I	–	–	12(6)	
	Hexane (AP)	*Yersinia enterocolitica ss. Entero colitica* (ATCC 23715)	CC			20 (6)	
***Echinops*** **species**	**Extract(plant part)**	**Strain (ID)**	**Type**	**MIC**	**MFC**	**Zone of inhibition (mm) (Conc.) (mg/mL)**	**Ref.**
*E. cephalotes*	Ethanol/Methanol/Water	*C. albicans*	I			18.9/16.3/18(7.8)	Heshmati et al., 2016
	Ethanol	*C. glabrata*	I			15.7(7.8)	
*E*. *ellenbeckii*	80% Methanol (L)	*C. albicans*	I	–	–	18.9 (10)	Hymete et al., 2005a
*E. kebericho*	Ethanol/methanol (R)	*A. flavus*	I	12.5/6.25 µg/mL	22.92/12.5 µg/mL	17.33/18.66 (0.08)	Ameya et al., 2016
		*C. albicans*	I	6.25/3.12 µg/mL	12.5/6.25 µg/mL	18.66/20.33 (0.08)	
*E. pinosissimus*	Methanol (AP)	*C. albicans*	S	–	–	18 (23)	Abd-Ellatif et al., 2011

AP, Aerial part; F, Fruit; L, Leaf; R, Root; St, Stem; WP, Whole plant; MDR, Multidrug resistant; I, Isolate; S, Standard. All studies resulting in MIC values over 1 mg were not included as such dosages cannot be applied in vivo.

Out of the tested strains, *M. tuberculosis* (H37Rv) showed higher sensitivity to the ether root extract of *E. giganteus* and methanolic extract of *E. amplexicaulis* Oliv. with MIC of 12 µg/mL and 32 µg/mL, respectively (Tekwu et al., 2012; Kevin et al., 2018). The methanolic root extract of *E. amplexicaulis* also showed a promising effect against a multidrug-resistant strain of *M. tuberculosis* with a MIC of 50 µg/mL (Kevin et al., 2018). The ethanolic root extract and essential oils obtained from *E. kebericho* Mesfin showed relatively strong effect against *Staphylococcus aureus* (Ameya et al., 2016) and *Klebsiella pneumoniae* (Belay et al., 2011). These results might justify the traditional application of *E. kebericho* in treating respiratory disease, toothache, and fever. The essential oil from *E. ritro* L. exhibited anti-bacterial effect and antibiofilm and disruption of the bacterial membrane were suggested as mechanisms of actions (Jiang et al., 2017).

Different extracts from members of the genus having anti-bacterial effect were analyzed for their chemical constituents. The unsaponifiable matter from the hexane extract of *E. spinosissimus* contained mainly taraxasterol, lupeol, pseudotaraxasterol, α-amyrin, β-amyrin, pseudotaraxasteryl acetate, lup-22(29)-en-3-yl acetate, β–sitosterol, and stigmasterol. The hexane extract showed anti-bacterial activity with MIC values of less than 125 µg/mL against different bacterial strains (*Bacillus amyloliquefaciens*, *Micrococcus luteus*, *Bacillus subtilis*, and *Salmonella enteric*) (Bouattour et al., 2016). Thiophens (**31**, **46**, **54**, and **59**) isolated from the root of *E. ritro* possessed anti-bacterial effect against *S. aureus* with a MIC value of 8 µg/mL. This was similar to the effect observed for the positive control, levofloxacin. The anti-bacterial effects of thiophenes **31**, **46**, **55**, **57**, and **59** against *Escherichia coli* with a MIC of 64, 32, 64, 64, and 8 µg/mL, respectively, were also described (Li et al., 2019).

In addition to those described in **Table 3**, the root extract of *Echinops* spp from Ethiopia showed anti-bacterial activity through growth inhibition (Ashebir and Ashenafi, 1999). The study did not delineate the specific name of the plant, MIC/MBC, and zone of inhibitions which makes it challenging to compare with other study results. Methanolic extract of the whole plant of *E. polyceras* improved the effect of tetracycline on resistant strains of *Pseudomonas aeruginosa* (Aburjai et al., 2001). The effect of the plant without tetracycline however was not studied. The leaf and flower extracts of *E. viscosus* subsp. bithynicus were described to have anti-bacterial properties against *E. coli*, *Micrococcus luteus*, *S. aureus*, *Mycobacterium smegmatis*, *P. aeruginosa*, *Enterobacter cloacae*, and *Bacillus megaterium*. Even though the concentration of the extracts is not well defined in the study, the flower extract of *E. microcephalus* has been reported to have greater zone of inhibition than the standard drug, vancomycin (30 µg/disc) (Toroğlu et al., 2012).

Most of the anti-fungal studies on the genus revealed that the extracts/isolated compounds were effective mainly against *Candida albicans* with the most potent effect observed for the root methanolic extract of *E. kebericho* (MIC = 3.12 µg/mL) (Ameya et al., 2016).

Thiophenes (**1**, **2, 5**, **18**, **22**, **23**, **31**, **38**, and **39**) from *E*. *ritro* have been described to possess significant anti-fungal activity against different fungal isolates. The most active thiophenes were **1** (IC_50_ = 4.2 µM) against *Colletotrichum gloeosporioides*, **2** (IC_50_ = 1.9 µM), and 5 (IC_50_ = 1.1 µM) against *C. fragariae* (Fokialakis et al., 2006a). A recent study also showed that thiophenes (**31**, **46**, and **55)** isolated from *E. ritro* exhibited anti-fungal effect against *C. albicans* with the MIC of 64, 32, and 64 µg/mL, respectively (Li et al., 2019). The anti-fungal activity of extracts obtained from *E. viscosus* subsp. bithynicus and *E. microcephalus* leaves and flowers were found to be active against *Saccharomyces cerevisiae*, *Rhodotorula rubra*, *Mucor pusillus*, and *Kluyveromyces fragilis* (Toroğlu et al., 2012).

### Effect on Cancer Cell Lines

The traditional use of the genus *Echinops* in the treatment of cancer is not common nevertheless the species in this genus were explored for cytotoxic activity. The methanolic extract of *E*. *kotschyi* Boiss. against MOLT-4 and K562 cancer cell lines (Afshaki et al., 2012) and essential oils obtained from *E. kebericho*, which consist of 43 compounds predominantly dehydrocostus lactone, showed cytotoxic activity against human monocytic leukemia cell line (THP-1) with an IC_50_ value of 0.4 µg/L (Tariku et al., 2011).

Four thiophens isolated from *E. latifolius* Tausch., 5-(3,4-dihydroxybut-1-ynyl)-2,2'-bithiophene (**15**), 5-(4-hydroxy-1-butynyl)-2,2'-bithiophene (**31**), 5-{4-[4-(5-pent-1,3-diynylthiophene-2-yl)-but-3-yny}-2,2'-bithiophene (**40**), and 5-(4-hydroxybut-1-one)-2,2'-bithiophene (**41**) were tested against human malignant melanoma (A375-S2) and human cervical carcinoma (HeLa) cell lines. The four compounds displayed cytotoxic activity and the effect was more when the mixture of cell lines and compounds were exposed to ultraviolet A (UVA) light for 30 min. The effects of the four compounds were higher against HeLa cell line with IC_50_ values of 5.2, 10.2, 3.1, and 6.5 µmol/L, respectively (Wang et al., 2007).

Jin et al. (2008) illustrated the *in vitro* cytotoxic activity of the dichloromethane fraction of the crude ethanolic root extract of *E*. *grijisi* and thiophenes (**1**, **2**, **9**, **18**, **23**, **34**, **39**, and **42**) isolated from this fraction. The fraction, as well as the isolated compounds showed different effects towards human hepatocarcinoma (HepG2 and MFC-7), human acute myeloid leukemia (HL-60), and human chronic myelogenous leukemia (K562) cell lines. The highest activities were observed for the dichloromethane fraction against HL-60 (IC_50_ = 5 µg/mL), 5-(4-isovaleroyloxybut-1-ynyl)-2,2'-bithiophene (**18**) against HepG2 (IC_50_ = 2 µg/mL), 5-(3-acetoxy-4-isovaleroyloxybut-1-ynyl)-2,2′-bithiophene (**34**) against HepG2 and K562 (IC_50_ = 1.8 and 7 µg/mL), and 5-(prop-1-ynyl)-2-(3,4-diacetoxybut-1-ynyl)-thiophene (**42**) against HL-60 (IC_50_ = 8 µg/mL). The dichloromethane fraction was tested in mice and did not show anti-tumor effect.

Similarly, Zhang et al. (2009) evaluated the cytotoxic effect of thiophenes isolated from *E. grijisii* on human cancer cell lines, HL60 and K562. Significantly potent effect was achieved with **5** (IC_50_ = 0.23 and 0.47 µg/mL) and **14** (IC_50_ = 0.27 and 0.43 µg/mL) against HL60 and K562, respectively. The thiophenes showed better activity against HL-60. A compound isolated from the root of *E. grijsii*, 5-(5,6-dihydroxy-hexa-1,3-diynyl)-2-(prop -1-ynyl)-thiophene (**13**), possessed anti-proliferative activity against human colon cancer cells, SW620, SW480, and HCT116 with IC_50_ values of 19.5 µM, 10.5 µM, and 27.7 µM, respectively, at 24 h. The proposed mechanism of action for the thiophene (**13**) was mitochondrial-mediated apoptosis (Zhang and Ma, 2010; Xu et al., 2015).

The methanolic extract from the underground part of *E*. *giganteus* also exhibited cytotoxic activity with an IC_50_ values of 9.84, 6.68, and 7.96 µg/mL against prostate cancer (Mia PaCa2) and two leukemia cells (CCRF-CEM and CEM/ADR5000), respectively (Kuete et al., 2011). In addition, the crude extract showed strong activity against breast cancer (MDA-MB-231-pcDNA3) with an IC_50_ value of 4.17 µg/mL. The secondary metabolites (**5**, **97**, **126**, and **131**) from the methanolic extract of this plant were tested for their cytotoxic effect and showed lower effect than that of the crude extract (Kuete et al., 2013). In continuation of this study, 5-(3,4-dihydroxybut-1-ynyl)-2-(penta-1,3-diynyl)-thiophene (**14**), echinopsolide A (**98**), and tetrahydrofurano-ceramide (**150**) were isolated from *E*. *giganteus*. These three compounds tested against leukemia showed the highest activity on CCRF-CEM (IC_50_ values of 46.96, 36.78, and 9.83 µM, respectively) and CEM/ADR5000 (IC_50_ values of 21.09, 38.57, and 6.12 µM, respectively) cell lines (Sandjo et al., 2016).

Macrochaetosides A and B (**80** and **81**) and cyclostenol (**104**), isolated from aerial parts of *E. macrochaetus* Boiss., were tested for their cytotoxic activity. The activity was observed on cell lines of breast adenocarcinoma (MCF-7) (IC_50_ = 2.1 and 0.18 μM), human hepatocellular carcinoma (HepG2) (IC_50_ = 2.9 and 3.3 μM), and colorectal adenocarcinoma (HCT-116) (IC_50_ = 3.6 and 2.3 μM) for cyclostenol and macrochaetosides A, respectively. Macrochaetoside B only showed a cytotoxic activity against MCF-7 with an IC_50_ of 6.9 μM (Zamzami et al., 2019).

The vehicle used to dissolve the compounds for the cytotoxicity study is not mentioned in some of the reports (Sandjo et al., 2016; Zamzami et al., 2019). In one study, α-terthiophene (**2**) was used as a positive control against A375-S2 (IC_50_ = 10.6 µmol/L) and HeLa (IC_50_ = 6.3 µmol/L) cell lines (Wang et al., 2007). Similarly α-terthiophene showed cytotoxic effect towards K562 (IC_50_ = 50 µg/mL) and HepG2 (IC_50_ = 10µg/mL) (Jin et al., 2008).

The above-described effects on cancer cell lines could be mainly due to thiophenes. Terpenoids and ceramides were the other secondary metabolites having a cytotoxic effect. Among the cell lines tested, leukemia cell lines were comparatively more sensitive in which 5-(4-hydroxybut-1-ynyl)-2-(pent-1,3-diynyl)-thiophene (**5**) showed the most potent effect.

Even though the extracts and isolated compound from the genus showed promising effects against different cancer cell lines, the effects are ought to be further investigated using *in vivo* models.

### Hepato-Protective and Anti-Oxidant Activities

Members of the genus *Echinops* were also shown to have hepatoprotective and anti-oxidant activities. Most of the studies were conducted in carbon tetrachloride (CCl_4_)-induced liver damage, in which biomarkers of liver function like aspartate aminotransferase (AST) and alanine aminotransferase (ALT) were measured.

The methanolic root extract, as well as *n-*butanol and aqueous fractions of *E. grijsii, showed* hepatoprotective activity in CCl_4_-induced liver damage in rats. The effect was prominent in the aqueous and butanol fractions, at a dose of 300 mg/kg, that markedly decreased AST and ALT levels (Lin et al., 1993). A study conducted by Eram et al. (2013) in CCl_4_-intoxicated rabbits justified the traditional claim of *E. echinatus* to treat jaundice (Gupta et al., 2010). The ethanolic aerial parts extract of *E. echinatus* at 500 and 750 mg/kg resulted in a significant decrease of ALT and AST, of which the lower dose (500 mg/kg) showed a higher effect (Eram et al., 2003). As presented in **Table 1**, flavonoids were isolated from the root of *E. grijsii* and the whole plant of *E. echinatus*. These might be responsible for the hepatoprotective effects of the extracts (Wang et al., 2015; Zang et al., 2017) and further investigations are required on phytoconstituents of the plants.

The hepatoprotective effect of compounds isolated from members of the genus *Echinops* was also investigated along with crude extracts. The protective effects of *E*. *galalensis* Schweinf. as well as isolated compounds β-sitosterol (**89**), apigenin-7-*O*-β-D-glucoside (**109**), 3β-acetoxy-taraxast-12,20(30)-diene-11α-21α–diol (**100**), α-amyrin (**101**), erythrodiol (**102**), lup-20(29)-ene-1,3-diol (**103**), and dicaffeoyl-quinic acid derivatives (**135-138**) on human hepatoma cell line (Huh7) have also been established. The highest protection was exhibited by **100**, **102**, and **103** and they significantly decreased the level of ALT. Except for the crude extract, all the tested samples decrease the level of AST and **89**, **101**, and **135** showed the highest effect (Abdallah et al., 2013). According to Abdallah et al. (2013), the protective effect of the extract and isolated compounds was suggested to be partly due to anti-oxidant effects of the samples.

Methotrexate-induced hepatotoxicity was also used to evaluate the hepatoprotective effect of some of the plants. Using this model, the protective effect of ethanolic aerial part extract and flavonoid fraction of *E. heterophyllus* P.H. Davis was established in rabbits. The crude ethanolic extract (250 mg/kg) significantly decreased the serum proteins, liver enzymes, and oxidative stress markers than the flavonoid fraction (Abdulmohsin et al., 2019).

In liver diseases, excessive oxidative stress undoubtedly contributes to the progression and pathological expression of the disease and serves as a prognostic indicator (Zhu et al., 2012). The methanolic root extract of *E*. *giganteus* showed *in vitro* free radical scavenging effect with 12.54 mg equivalent weight of trolox per 100 g (Bouba et al., 2010). The aqueous extracts of *E*. *ritro*, *E*. *tournefortii* Ledeb. possessed 2,2-diphenyl-1-picrylhydrazyl (DPPH) scavenging effect with inhibitions more than 80% and 70%, respectively, at 1 mg/mL (Aydın et al., 2016). A study that compared different types of extraction methods on antioxidant activity reported that hot extraction using methanolic-ethyl acetate of *E. persicus* showed higher *in vitro* free radical scavenging effect (89.14%) against DPPH (Mohseni et al., 2017). The free radical scavenging effect of crude seed and leaf extracts *E. orientalis* Trautv. as well as isolated compounds β-sitosterol (**89**) and 1-methylquinolin-4(*1H*)-one (**139**) from seeds and apigenin-7-*O*-β-D-glucoside (**109**) and apigenin-7-*O*-(6"-trans-*p-*coumaroyl-β-D-glucopyranoside (**124**) from leaf methanolic extract was demonstrated. The extracts showed a significant effect (> 60% at 40 µg/mL) while the effect of the isolated compounds was not significant against 2,2-diphenyl-1-picrylhydrazyl (DPPH). However, the two flavonoids (**109** and **124**) showed better scavenging effect towards 3-ethylbenzothiazoline-6-sulfonic acid (ABTS) radical cation than the extracts and the other two compounds (**89** and **139**), with IC_50_ of 3 and 5 µg/mL (Erenler et al., 2014).

Active cell cultures of human peripheral blood mononuclear cells were also used to evaluate the anti-oxidant effect of aqueous methanolic extract of *E. albicaulis* aerial parts. The study showed that the active oxygen species (ROS) generation in the cells was significantly reduced at concentrations of 1, 20, and 50 mg/mL of the extract; however, the extract induced overproduction ROSs at higher concentrations (Kiyekbayeva et al., 2017).

Regardless of the effects described, the anti-oxidant activity evaluations are not still sufficient. In most of the reports the IC_50_ value for the *in-vitro* anti-oxidant effect are not mentioned. No single *in vivo* anti-oxidant model was employed. In some of the hepatoprotective effect studies standard drugs were not utilized and comparison was made only with the negative control (**Table 5**). The hepatoprotective effect of traditionally used plant, *E. spinosus* L. (Akdime et al., 2015), has not been scientifically investigated yet.

### Anti-Inflammatory, Analgesic, Anti-Pyretic, and Wound Healing Activities

Traditionally, members of the genus *Echinops* are documented to have been used to treat inflammation, pain, and fever. Accordingly, several species have been explored for anti-inflammatory, analgesic, and anti-pyretic activities.

The whole plant ethanolic extract of *E. echinatus* showed anti-inflammatory activity against carrageenan and formaldehyde induced edema in rats with inhibitions of 67.5% and 51.8% at a dose of 800 mg/kg administered intraperitoneally and orally, respectively (Singh et al., 1989). A triterpenoid isolated from this plant, taraxasterol acetate (**88**), showed anti-inflammatory activity on carrageenan-induced pedal edema in rats with the highest inhibition of 68.3% and 63.2% at 200 mg/kg administered by the intraperitoneal and oral route, respectively (Sing et al., 1991). Flavanone glycoside, 5,7-dihydroxy-8,4'-dimethoxyflavanone-5-O-α-L-rhamno-pyranosyl-7-O-β-D-arabinopyranosyl (1→4)-O-β-D-glucopyranoside (**125**) isolated from *E. echinatus*, showed anti-inflammatory activity (Yadava and Singh, 2006). The methanolic root and aerial part extract of the plant showed analgesic properties in both hotplate and tail immersion models. The aerial part exhibited the highest activity by increasing the reaction time in both models to 7.99 and 7.77 sec, respectively, at 500 mg/kg, and it was comparable with the standard drug, pentazocine (Patel et al., 2011b). The ethanolic leaf and stem extract of *E. echinatus* showed antipyretic effect at a dose of 750 mg/kg in rabbits (Alam et al., 2016).

The methanolic root extracts of *E. spinosus*, *E. grijissi*, and *E. latifolius* exhibited significant anti-inflammatory activity (Lin et al., 1992; Rimbau et al., 1999). The ethyl acetate, chloroform, and n-hexane fractions obtained from the crude extract of *E. grijissi* showed significant anti-inflammatory activities in carrageenan-induced edema in rats, of which the chloroform fraction, at a dose of 300 mg/kg, exhibited inhibitory effect (56.7%) higher than that of indomethacin (Lin et al., 1992). Flavonoids, extracted from *E. latifolius*, were tested on rheumatoid arthritis using rats and inhibited the synovium proliferation through fibroblast-like synoviocytes apoptosis at 150 mg/kg (Miao et al., 2015).

A study was conducted to evaluate the anti-inflammatory activity of compounds isolated from *E. latifolius*, 5-(1,2-dihydroxy-ethyl)-2-(*Z*)-hept-5-ene-1,3-diynylthiophene (**43**), 5-(1,2-dihydroxyethyl)-2-(*E*)-hept-5-ene-1,3-diynylthiophene (**44**), 6-methoxy-arctinol-b (**45**), arctinol-b (**46**), latifolanone A (**82**), arctinol (**47**), methyl [5'-(1-propynyf)-2,2'-bithienyl-5-yl] carboxylate (**48**), and atractylenolide-II (**83**) on inhibition of lipopolysaccharide (LPS)-induced nitric oxide (NO) production. In the order of presented compound names, thiophenic compounds numbered **43**-**46** inhibited the NO production with IC_50_ ranging from 12.8–42.7 µM, whereas the IC_50_ of **47**, **48**, and **83** were reported to be more than 100 µM (Jin et al., 2016).

The whole plant extract of *E. heterophyllus* and the alkaloidal faction facilitated epithelialization and left no scars in rabbits (Abdulrasool et al., 2013). This is the only wound healing activity reported on members of this genus although the dose, vehicle, and the standard drug are not described.

The *in vivo* anti-inflammatory effects of the genus seemed to be not promising since the plants resulted in an inhibition of edema at higher doses. In spite of the studies stated above, scientific data justifying the traditional claim of *E. bovei* (Boiss.) Maire., *E. cornigerus*, *E. kebericho*, *E. longifolius* A. Rich., *E. macrochaetus*, and *E. spinosissimus* to treat rheumatism and pain are not provided yet.

### Anti-Protozoal and Anti-Helmentic Activities

As presented in **Table 3**, *E. hoehnelii* Schweinf. and *E. kebericho* have been used in traditional treatment of malaria. These plants along with other species showed anti-malarial activity.

Aqueous extract of the aerial parts of *E*. *polyceras* exhibited strong (96%) *in vitro* growth inhibitory activity against *Plasmodium falciparum*. Nevertheless, the concentration of the extract used for the test and the standard drug used as positive control has not been reported (Sathiyamoorthy et al., 1999). A study on 70% ethanolic root extract *E. kebericho* resulted in an inhibition of parasitemia by 57.3% at a dose of 500 mg/kg in mice against *Plasmodium berghei* (Toma et al., 2015). A recent study conducted on the 70% methanolic extract from roots of *E. kebericho* exhibited 49.5% of inhibition at 1000 mg/kg in mice (Biruksew et al., 2018). This might suggest that the potency of *E. kebericho* extract could be dependent on the extraction solvent.

Dichloromethane faction of the 80% methanolic extract of *E*. *hoehnelii*, and thiophens (5-(penta-1,3-diynyl)-2-(3-chloro-4-acetoxy-but-1-ynyl)-thiophene (**10**), and 5-(penta-1,3-diynyl)-2-(3,4-dihydroxybut-1-ynyl)-thiophene (**14**)) possessed anti-malarial activity. The two compounds showed parasitemia inhibition of 32.7% and 50.2% at a dose of 100 mg/kg, respectively, against *P. berghei* in mice (Bitew et al., 2017).

Different studies showed that essential oils possess strong anti-protozoal effects. The essential oil isolated from *E. kebericho* displayed a strong activity against two *Leishmania* strains (*L*. *aethiopica* and *L*. *donovani)* with an EC_50_ values of 0.24 and 0.5 µg/mL (Tariku et al., 2011). Essential oil obtained from *E. giganteus* had anti-protozoal effect against *Trypanosoma brucei* with an IC_50_ of 10.5 µg/mL and GC-MS analysis of the oil revealed the presence of modheph-2-ene, presilphiperfolan-8-ol, presilphiperfol-7-ene, cameroonan-7-α-ol, and (*E*)-caryophyllene as the main constituents of the oil (Kamte et al., 2017).

The anti-helminthic effects of members of the genus were also described. The root 80% methanolic extract of *E*. *kebericho* showed higher anti-helmentic effect (LD_50_ = 57µg/mL) than niclosamide (LD_50_ = 84.5 µg/mL) against earthworms (Hymete and Kidane, 1991). The root 80% methanolic extracts of *E*. *ellenbeckii* as well as *E*. *longisetus* A. Rich. were active against earthworms with 100% mortality at 500 µg/mL (Hymete et al., 2005a). Essential oil from the root of *E*. *kebericho* showed lethal effect (81.8%) at a concentration of 1% (v/v) towards *Haemonchus contortus* (Hussien et al., 2011).

### Effects on Insects and Termites

The leaves of *Echinops* spp, which are commonly known as “Kebericho” in Ethiopia, had a mosquito repellant effect against *Anopheles arabiensis* with the effectiveness of 92.47% as a smoke (Karunamoorthi et al., 2008).

The activity of thiophenes (**2**, **8**, **9**, **10**, **14**, **15, 39**, **51**, and **52**) isolated from *E. transiliensis* Golosk. against *Aedes aegypti* was reported and the toxic effect increased with the number of thiophene moieties in the molecule. Strong activity was observed for 2''-terthiophene (**2**) with an LC_50_ value of 0.16 µg/mL (Nakano et al., 2014). Similarly, the root extract of *E. grijsii* possessed significant larvicidal activity against *Aedes albopictus*, *Anopheles sinensis*, and *Culex pipienspallens* with LC_50_ values of 2.65, 3.43, and 1.47 µg/mL, respectively.

Bioactivity-directed chromatographic separation of the essential oil obtained from *E. grijsii* led to the isolation of thiophenes. The larvicidal effects of the isolated compounds, 5-(3-buten-1-yn-1-yl)-2,2′-bithiophene (**1**) (LC_50_ 0.34, 1.36, and 0.12 µg/mL), α-terthienyl (**2**) (LC_50_ 1.41, 1.79, and 1.38 µg/mL), and 5-(4-isovaleroyloxybut-1-ynyl)-2,2'-bithiophene (**18**) (LC_50_ 0.45, 5.36, and 0.33 µg/mL) against the three organisms mentioned above was described (Zhao et al., 2017). On the contrary, the larvicidal activity of essential oils from *E. giganteus* against *Culex quinquefasciatus* was relatively low (LC_50_ = 227.4 μL/L) (Pavela et al., 2016).

Fokialakis et al. (2006b) evaluated the termicidal effect of eight thiophenes (**1**, **2**, **5**, **10**, **18**, **23**, **31**, and **39**) isolated from *E. ritro*, *E. spinosissimus*, *E. albicaulis*, and *E. transiliensis* on *Coptotermes formosanus*. The study revealed that all the thiophenes showed termicidal activity and 100% morality was observed after application of 5-(3-buten-1-ynyl)-2,2-bithiophene (**1**) and 2''-terthiophene (**2**) for 9 days at 2% and 1% (w/w), respectively. However, the exact concentrations of the compounds were not mentioned.

### Effects on the Reproductive System

A number of species have been used for the management of various reproductive health problems (**Table 1**). In spite of the traditional claims, only *E*. *echinatus* has been evaluated for these biological activities.

Corresponding to its traditional use, the terpenoidal fraction from *E*. *echinatus* displayed anti-fertility properties at doses of 30 and 60 mg/kg in male rats (Padashetty and Mishra, 2007). Earlier studies also indicated that the root ethanolic extract of *E*. *echinatus* has anti-fertility properties through decrement in sizes of testes, epididymis, ventral prostate, vas deferens, and seminal vesicle at doses of 50, 100, and 200 mg/kg. In addition, the extract also decreased sperm motility and density with an inhibition of sepermatogenesis in rats (Chaturvedi et al., 1995). The butanol fraction of the root extract demonstrated a protective effect on testosterone-induced prostatic hyperplasia at a dose of 100 mg/kg in rats. The butanol fraction also showed better 5α-reductase inhibitory effect (IC_50__=_ 0.22 mg/mL) than of the crude extract and other fractions followed by the water soluble fraction (IC_50__=_ 0.43 mg/mL) (Agrawal et al., 2012). Similarly, the root petroleum ether extract of *E. echinatus* inhibited 5α-reductase. The enzyme plays an important role in the pathogenesis of benign prostatic hyperplasia (BPH), prostatic cancer, acne, alopecia, baldness in men, and hirsutism in women (Nahata and Dixit, 2014).

### Other Activities

A study showed that 5-(penta-1,3-diynyl)-2-(3,4-dihydroxybut-1-ynyl)-thiophene (**14**), isolated from the root of *E. grijsii*, has an induction effect on nicotinamide adenine dinucleotide phosphate (NAD(P)H): quinone oxidoreductase1 (NQO1), an enzyme that is involved in detoxification of toxic quinones. The induction effect was dose-dependent and the maximum effect was observed at a concentration of 40 μM and it was 3.1 folds of the control, 4'-bromoflavone (Shi et al., 2010). Similarly, compounds **5**, **9**, and **14**, from the root of *E. grijisii*, had a strong NQO1-inducing effect and the concentrations that caused a twofold induction were 1.86, 2.58, and 2.87 μg/mL, respectively. Compounds **5** and **14** were found to have an alkylating effect on cysteine residues in NF-κB (nuclear factor kappa-light-chain-enhancer of activated B cells) (Zhang and Ma, 2010).

The 70% hydro-alcoholic root extract of *E. echinatus* was reported to have significant anti-diabetic activity on alloxan-induced diabetic rats. The the extract treated animals (200 mg/kg) showed lower blood glucose level (164 mg/dL) compared to the negative control (277.6 mg/dL) after 21 days of treatment. In addition, the extract exhibited the ability to regenerate pancreatic islet cells and normal structure of glomeruli and proximal and distal convoluted tubules in kidneys (Fatima et al., 2017). Similarly, the methanolic root extract of *E. echinatus* exhibited a significant anti-diabetic effect at doses of 100 and 200 mg/kg on alloxan induced diabetic rats. The extract was also able to decrease serum cholesterol, serum triglyceride, serum low-density lipoprotein, serum very low-density lipoprotein, and serum alkaline phosphate significantly while it increased high-density lipoproteins (Sarvaiya et al., 2017).

The molluscicidal activities of 80% methanolic root extracts of *E*. *ellenbeckii* and *E. longisetus* with a 100% mortality rate at 20.25 and 45 µg/mL, respectively, was described (Hymete et al., 2005a). The pancreatic amylase inhibitory activity (> 75%) of aqueous root extract of *E*. *giganteus* was reported although the exact concentration of the extract was not mentioned (Etoundi et al., 2010). The latex of *E*. *persicus* at 500 mg/kg resulted in lower number and level of stomach ulcer compared to the negative control in rats (Rad et al., 2010). The methanolic extract of root and aerial parts of *E*. *echinatus* significantly increased urine volume and excretion at doses of 250 and 500 mg/kg (Patel et al., 2011a). The immunomodulating activity of aerial parts methanolic extract of *E. lasiolepis* Bunge has been reported. The extract at different concentrations (0.1, 1, 10, 100, and 200 µg/mL) inhibited peripheral blood mononuclear cells (PBMCs) proliferation of which 1 µg/mL showed optimum proliferation (30.66%) (Asadi et al., 2014).

Biological effects evaluated on genus *Echinops* and the doses with maximum effect are summarized in **Tables 3**–**5**.

**Table 4:** *In vitro* cytotoxic effect of members of the genus *Echinops*.

Plant/fraction/compound name (Plant)	Cell line	Positive control	Negative control	IC_50_	References
Essential oils(*E. kebericho*)	Human monocytic leukemia (THP-1)	Amphotericin B	1% DMSO	0.4 µg/mL	Tariku et al., 2011
**15** (*E. latifolius*)	Human cervical carcinoma (HeLa)	α-terthienyl	DMSO	5.2 µmol/L	Wang et al., 2007
**31** (*E. latifolius*)	HeLa	α-terthienyl	DMSO	10.2 µmol/L	Wang et al., 2007
**40** (*E. latifolius*)	HeLa	α-terthienyl	DMSO	3.1 µmol/L	Wang et al., 2007
**41** (*E. latifolius*)	HeLa	α-terthienyl	DMSO	6.5 µmol/L	Wang et al., 2007
Dichloromethane fraction (*E*. *grijisi*)	Human acute myeloid leukemia (HL-60)	Platinol	DMSO	5 µg/mL	Jin et al., 2008
**18** (*E*. *grijisi*)	Human hepatocarcinoma (HepG2)	Adriamycin	DMSO	2 µg/mL	Jin et al., 2008
**34** (*E*. *grijisi*)	HepG2	Adriamycin	DMSO	1.8 µg/mL	Jin et al., 2008
**34** (*E*. *grijisi*)	Human chronic myelogenous leukemia (K562)	Adriamycin	DMSO	7 µg/mL	Jin et al., 2008
**42** (*E*. *grijisi*)	HL-60	Platinol	DMSO	8 µg/mL	Jin et al., 2008
**5** (*E*. *grijisi*)	HL-60	Platinol	DMSO	0.23 µg/mL	Zhang et al., 2009
**5** (*E*. *grijisi*)	K562	Adriamycin	DMSO	0.47µg/mL	Zhang et al., 2009
**14** (*E*. *grijisi*)	HL-60	Platinol	DMSO	0.27 µg/mL	Zhang et al., 2009
**14** (*E*. *grijisi*)	K562	Adriamycin	DMSO	0.43 µg/mL	Zhang et al., 2009
**13** (*E*. *grijisi*)	Colon cancer (SW480)	4′-Bromoflavone	DMSO	19.5 µM	Zhang and Ma, 2010
**13** (*E*. *grijisi*)	Colon cancer (SW480)	4′-Bromoflavone	DMSO	10.5 µM	Zhang and Ma, 2010
**13** (*E*. *grijisi*)	Colon cancer (HCT116)	4′-Bromoflavone	DMSO	27.7µM	Zhang and Ma, 2010
*E*. *giganteus*	Prostate cancer (Mia PaCa2)	Doxorubicin	DMSO	9.84 µg/mL	Kuete et al., 2011
*E*. *giganteus*	Leukemia (CCRF-CEM)	Doxorubicin	DMSO	6.68 µg/mL	Kuete et al., 2011
*E*. *giganteus*	Leukemia (CEM/ADR5000)	Doxorubicin	DMSO	7.96 µg/mL	Kuete et al., 2011
**14** (*E*. *giganteus*)	CCRF-CEM	Doxorubicin	NM	46.96 µM	Sandjo et al., 2016
**14** (*E*. *giganteus*)	CEM/ADR5000	Doxorubicin	NM	21.09 µM	Sandjo et al., 2016
**98** (*E*. *giganteus*)	CCRF-CEM	Doxorubicin	NM	36.78 µM	Sandjo et al., 2016
**98** (*E*. *giganteus*)	CEM/ADR5000	Doxorubicin	NM	38.57 µM	Sandjo et al., 2016
**150** (*E*. *giganteus*)	CCRF-CEM	Doxorubicin	NM	9.83 µM	Sandjo et al., 2016
**150** (*E*. *giganteus*)	CEM/ADR5000	Doxorubicin	NM	6.12 µM	Sandjo et al., 2016
**80** (*E. macrochaetus*)	Breast adenocarcinoma (MCF-7)	Doxorubicin	NM	0.18 µM	Zamzami et al., 2019
**80** (*E. macrochaetus*)	HepG2	Doxorubicin	NM	3.3 µM	Zamzami et al., 2019
**80** (*E. macrochaetus*)	MCF-7	Doxorubicin	NM	2.1 µM	Zamzami et al., 2019
**104** (*E. macrochaetus*)	HepG2	Doxorubicin	NM	2.9 µM	Zamzami et al., 2019
**104** (*E. macrochaetus*)	MCF-7	Doxorubicin	NM	6.9 µM	Zamzami et al., 2019

DMSO, Dimethyl sulfoxide; NM, Not mentioned.

**Table 5:** Other biological effects of members of the genus *Echinops*.

Hepatoprotective and antioxidant activities						
Plant/compound name (Plant)	Effects ^Model^	Positive control	Negative control	Dose/Concentration (Rout of administration)	Activity	References
**100**, **102**, and **103** (*E*. *galalensis*)	Hepatoprotective ^2^	Silymarin	PBS	100 mg/ml	↓ ALT	Abdallah et al., 2013
**100**, **102**, and **103** (*E*. *galalensis*)	Hepatoprotective ^2^	Silymarin	PBS	100 mg/ml	↓ AST	Abdallah et al., 2013
**109** (*E. orientalis*)	Anti-oxidant ^2^	Trolox	NM		IC_50_ = 3 µg/mL	Erenler et al., 2014
**131** (*E. orientalis*)	Anti-oxidant ^2^	Trolox	NM		IC_50_ = 5 µg/mL	Erenler et al., 2014
*E. albicaulis*	Anti-oxidant ^2^	N-acetylcysteine	NM	1, 20, and 50 mg/mL	↓ Generation of ROSs	Kiyekbayeva et al., 2017
*E. echinatus*	Hepatoprotective ^1^	Silymarin	Normal saline	500 mg/kg (p.o.)	↓ AST and ALT	Eram et al., 2013
*E*. *giganteus*	Anti-oxidant ^2^	Trolox	Distilled water		12.54 mg equivalent weight of trolox per 100 g	Bouba et al., 2010
*E. grijsii*	Hepatoprotective ^1^	NM	Normal saline	300 mg/kg (p.o.)	↓ AST and ALT	Lin et al., 1993
*E. heterophyllus*	Hepatoprotective ^1^	NM	Distilled water	250 mg/kg (p.o.)	↓ AST, ALT, and Aalkaline phosphatase(ALP)	Abdulmohsin et al., 2019
*E. orientalis*	Anti-oxidant ^2^	Trolox	NM	40 µg/mL	> 60%	Erenler et al., 2014
*E. persicus*	Anti-oxidant ^2^	NM	Methanol		89.1%	Mohseni et al., 2017
*E*. *ritro*	Anti-oxidant ^2^	BHT (Dibutylhydroxytoluene)	Distilled water	1 mg/mL	> 80%	Aydın et al., 2016
*E*. *tournefortii*	Anti-oxidant ^2^	BHT	Distilled water	1 mg/mL	> 70%	Aydın et al., 2016
**Anti-inflammatory, analgesic, anti-pyretic and wound healing activities**						
**132** (*E. echinatus)*	Anti-inflammatory ^1^	Phenylbutazone	1% gumacacia	200 mg/kg (i.p.)	Inh = 68.3%	Singht et al., 1991
**43** (*E. latifolius*)	Inhibition of LPS-induced NOproduction ^2^	Aminoguanidine and Indomethacin	NM		IC_50_ = 12.8 µM	Jin et al., 2016
**44** (*E. latifolius*)	Inhibition of LPS-induced NOproduction ^2^	Aminoguanidine and Indomethacin	NM		IC_50_ = 28.2 µM	Jin et al., 2016
**45** ( *E. latifolius*)	Inhibition of LPS-induced NOproduction ^2^	Aminoguanidine and Indomethacin	NM		IC_50_ = 30.9 µM	Jin et al., 2016
**46** (*E. latifolius*)	Inhibition of LPS-induced NOproduction ^2^	Aminoguanidine and Indomethacin	NM		IC_50_ = 48.6 µM	Jin et al., 2016
**47** (*E. latifolius*)	Inhibition of LPS-induced NOproduction ^2^	Aminoguanidine and Indomethacin	NM		IC_50_ = > 100 µM	Jin et al., 2016
**48** (*E. latifolius*)	Inhibition of LPS-induced NOproduction ^2^	Aminoguanidine and Indomethacin	NM		IC_50_ = > 100 µM	Jin et al., 2016
**83** (*E. latifolius*)	Inhibition of LPS-induced NOproduction ^2^	Aminoguanidine and indomethacin	NM		IC_50_ = > 100 µM	Jin et al., 2016
Chloroform fraction (*E. grijissi*)	Anti-inflammatory	Indomethacin	Normal saline	300 mg/kg (i.p.)	Inh = 56%	Lin et al., 1992
*E. echinatus*	Anti-inflammatory ^1^	Phenylbutazone	1% gumacacia	800 mg/kg (i.p.)	Inh = 67.5%	Singh et al., 1989
*E. echinatus*	Analgesic ^1^	Pentazocine	Distilled water	500 mg/kg (p.o)	↑ Reactionary time	Patel et al., 2011b
*E. echinatus*	Antipyretic ^1^	Paracetamol	NM	750 mg/kg	↓ Rectal temperature	Alam et al., 2016
*E. heterophyllus*	Wound healing ^1^	NM	NM	NM	Facilitated epithelialization	Abdulrasool et al., 2013
Flavonoids (*E. latifolius*)	Inhibition of rheumatoid arthritis ^1^	NM	Phosphate-buffered saline (PBS)	50, 100 and 150 mg/kg	↓ Arthritis and paw swelling score	Miao et al., 2015
**Anti-protozoal and anti-helmentic activities**						
**10** (*E*.*hoehnelii*)	Anti-malarial ^1^	Chloroquine	7% Tween 80/3% ethanol	100 mg/kg	Inh = 32.7%	Bitew et al., 2017
**14** (*E*.*hoehnelii*)	Anti-malarial ^1^	Chloroquine	7% Tween 80/3% ethanol	100 mg/kg	Inh = 50.2%	Bitew et al., 2017
*E. ellenbeckii*	Anti-helmentic ^2^	Niclosamide	Tap water	500 µg/mL	Mortality rate = 100%	Hymete et al., 2005a
*E. kebericho*	Anti-malaria ^1^	Chloroquine	(3% of Tween 80	500 mg/kg	Inh = 57.3%	Toma et al., 2015
*E. kebericho*	Anti-helmentic ^2^	Niclosamide	Tap water		LD_50_ = 57 µg/mL	Hymete and Kidane 1991
*E. longisetus*	Anti-helmentic ^2^	Niclosamide	Tap water	500 µg/mL	Mortality rate = 100%	Hymete et al., 2005a
*E*. *polyceras*	Anti-malarial ^2^	NM	Distilled water	0.2% (w/v)	Inh = 96%	Sathiyamoorthy et al., 1999
Essential oil (*E. giganteus*)	Anti-trypanosomal ^2^	Suramin	DMSO		IC_50__=_ 10.5 µg/mL	Kamte et al., 2017
Essential oil (*E. kebericho*)	Anti-leishmanial ^2^	Amphotericin B	1% DMSO		EC_50__=_ 0.24 µg/mL	Tariku et al., 2011
Essential oil (*E*. *kebericho*)	Anti-helmentic ^2^	Thiabendazole	0.5% Tween 80 in PBS	1% (v/v)	Inh = 81.8%	Hussien et al., 2011
**Effects on insects and termites**						
**1** (*E. grijsii*)	Larvicidal ^2^	Rotenone	0.25% Tween 40		LC_50__=_ 0.12 µg/mL	Zhao et al., 2017
**1**, **2** (*E. ritro* and *E. spinosissimus*)	Termicidal ^2^	NM	Distilled water	1% (w/w)	Mortality rate = 100%	Fokialakis et al., 2006b
**10** (*E. transiliensis*)	Larvicidal ^2^	Permethrin	DMSO		LC_50__=_ 14.71 µg/mL	Nakano et al., 2014
**14** (*E. transiliensis*)	Larvicidal ^2^	Permethrin	DMSO		LC_50__=_ 12.45 µg/mL	Nakano et al., 2014
**15** (*E. transiliensis*)	Larvicidal ^2^	Permethrin	DMSO		LC_50__=_ 9.89 µg/mL	Nakano et al., 2014
**18** (*E. grijsii*)	Larvicidal ^2^	Rotenone	0.25% Tween 40		LC_50__=_ 0.33 µg/mL	Zhao et al., 2017
**2** (*E. grijsii*)	Larvicidal ^2^	Rotenone	0.25% Tween 40		LC_50__=_ 1.38 µg/mL	Zhao et al., 2017
**2** (*E. transiliensis*)	Larvicidal ^2^	Permethrin	DMSO		LC_50__=_ 0.16 µg/mL	Nakano et al., 2014
**39** (*E. transiliensis*)	Larvicidal ^2^	Permethrin	DMSO		LC_50__=_ 4.22 µg/mL	Nakano et al., 2014
**51** (*E. transiliensis*)	Larvicidal ^2^	Permethrin	DMSO		LC_50__=_ 7.45 µg/mL	Nakano et al., 2014
**52** (*E. transiliensis*)	Larvicidal ^2^	Permethrin	DMSO		LC_50__=_ 19.97 µg/mL	Nakano et al., 2014
**8** (*E. transiliensis*)	Larvicidal ^2^	Permethrin	DMSO		LC_50__=_ 18.55 µg/mL	Nakano et al., 2014
**9** (*E. transiliensis*)	Larvicidal ^2^	Permethrin	DMSO		LC_50__=_ 17.95 µg/mL	Nakano et al., 2014
Butanol fraction (*E. echinatus*)	Anti- hyperplasia ^1^	Finasteride	2% Tween 80	50, 100, and 200 mg/kg (p.o.)	↓ Prostatic/body weight ratio	Agrawal et al., 2012
Butanol fraction (*E. echinatus*)	5α-reductase inhibitory effect ^2^	Finasteride	Ethanol		IC_50__=_ 0.22 mg	Agrawal et al., 2012
*E. echinatus*	Anti-fertility ^1^	NM	Distilled water	50, 100, and 200 mg/kg	↓ sizes of testes, epididymis, ventral prostate, vas deferens, and seminal vesicle	Chaturvedi et al., 1995
Essential oil (*E. giganteus*)	Larvicidal ^2^	NM	DMSO		LC_50__=_ 227.4 µL/L	Pavela et al., 2016
**Effects on the reproductive system**						
Terpenoidal fraction (*E. echinatus*)	Effect on male reproductive parameters ^1^	NM	1% Tween 80	60 mg/kg (p.o.)	↓ Seminiferous tubular diameter and germinal epithelial cell thickness	Padashetty and Mishra, 2007
**Other activities**						
**14** (*E. grijsii*)	NQO1 inducing activity ^2^	4'-Bromoflavone	NM	40 µM	Induction = 3.1X of the control	Shi et al., 2010
**14** (*E. grijsii*)	NQO1 inducing activity ^2^	4'-Bromoflavone	NM	2.87 µg/mL	Induction = 2X of the control	Zhang and Ma, 2010
**5** (*E. grijsii*)	NQO1 inducing activity ^2^	4'-Bromoflavone	NM	1.86 µg/mL	Induction = 2X of the control	Zhang and Ma, 2010
**9** (*E. grijsii*)	NQO1 inducing activity ^2^	4'-Bromoflavone	NM	2.58 µg/mL	Induction = 2X of the control	Zhang and Ma, 2010
*E. echinatus*	Anti-diabetic ^1^	Sitagliptin	Normal saline	200 mg/kg (p.o.)	↓ Blood glucose level	Fatima et al.2017
*E. echinatus*	Anti-diabetic ^1^	MetforminHCl	1% Tween 80 in saline	200 mg/kg (p.o.)	↓ Blood glucose level	Sarvaiya et al., 2017
*E*. *echinatus*	Diuretic	Furosemide	Normal saline	500 mg/kg (p.o.)	↑ Urine volume and electrolyte excretion	Patel et al., 2011a
*E. ellenbeckii*	Molluscicidal ^2^	NM	De-chlorinated tap water	20.25 µg/mL	Mortality rate = 100%	Hymete et al., 2005a
*E*. *giganteus*	Amylase inhibitory ^2^	NM	Distilled water	NM	> 75%	Etoundi et al., 2010
*E. lasiolepis*	Immunomodulating activity	NM	DMSO	1 µg/mL	Inhibited PBMCs proliferation	Asadi et al., 2014
*E. longisetus*	Molluscicidal ^2^	NM	De-chlorinated tap water	45 µg/mL	Mortality rate = 100%	Hymete et al., 2005a
*E*. *persicus*	Anti-ulcer	NM	Distilled water	500 mg/kg (p.o./i.p.)	↓ Number and level of stomach ulcer	Rad et al., 2010

DMSO, Dimethyl sulfoxide; NM, Not mentioned; p.o., Per os (Oral); i.p., intraperitoneal; ^1^, In vivo; ^2^, In vitro.

## Conclusion

The genus *Echinops* is well known for its use to treat pain and respiratory manifestations. The traditional claims were justified by different biological evaluations. Findings from *in vitro* studies indicated that members of the genus have a potential effect against different cancer lines, microbial strains, and insects. They also showed significant *in vivo* anti-inflammatory, analgesic, and hepatoprotective activities. Some of the extracts and isolated compounds showed promising effects. This includes the anticancer activity of compounds **5** and **14**, antioxidant potential of **109**, anti-leishmanial and anti-helmentic effects of *E. kebericho*, and the larvicidal effect of compound **1**. The safety and efficacy of secondary metabolites responsible for the *in vitro* effects of extracts/fractions should further be investigated in *in vivo* models. The most abundant bioactive secondary metabolites in members of the genus are thiophenes and terpenoids which are also mentioned as responsible for the cytotoxic effect observed. In the current review, it has been observed that the potential uses of the species in the removal of kidney stones and use to solve nerve-related problems have not been scientifically addressed yet. Investigation of the anti-microbial activity of isolated compounds seems to be limited. We believe this review will provide summarized information to the scientific community working on the genus.

## Author Contributions

HB developed concept of the review, conducted the literature review, extracted relevant information to the study, and drafted the manuscript. AH guided the literature search and edited the manuscript. Both authors have read and approved the manuscript.

## Conflict of Interest

The authors declare that the research was conducted in the absence of any commercial or financial relationships that could be construed as a potential conflict of interest.

## References

[B1] Abdallah H. M. Ezzat S. M. Dine R. S. Abdel-Sattar E. Abdel-Naim A. B. (2013). Protective effect of *echinops galalensis* against CCl_4_-induced injury on the human hepatoma cell line (Huh7). Phytochem. Lett. 6, 73–78. 10.1016/j.phytol.2012.10.01

[B2] Abd-Ellatif S. Abdel Rahman S. M. Deraz S. F. (2011). Promising antifungal effect of some folkloric medicinal plants collected from El-Hammam habitat, Egypt against dangerous pathogenic and toxinogenic fungi. J. Agric. Biol. Sci. 6, 25–32.

[B3] Abderrahim O. Martin G. J. Abdelaziz A. (2013). Botanical identification and ethno-medicinal uses of some underground part of medicinal plants collected and traded in Marrakech region. J. Med. Plants. Res. 7, 2165–2169. 10.5897/JMPR11.1597

[B4] Abdulrasool A. A. Fahmi Z. M. Khadeem E. J. (2013). Relative assess on wound healing and anti scar activity of crude *Echinops heterophyllus* extract and some of its bioactive fractions. Int. J. Pharm. Pharm. Sci. l5, 468–475.

[B5] Abdou Bouba A. Njintang Yanou N. Foyet H. Scher J. Montet D. Mbofung C. M. (2012). Proximate composition, mineral and vitamin content of some wild plants used as spices in Cameroon. Food Nutr. Sci. 3, 423–432. 10.4236/fns.2012.34061

[B6] Abdulmohsin H. Raghif A. A. Manna M. J. (2019). The protective effects of *echinops heterophyllus* extract against methotrexate-induced hepatotoxicity in rabbits. Asian. J. Pharm. Clin. Res. 12, 384–390. 10.22159/ajpcr.2019.v12i1.30213

[B7] Abegaz B. M. (1991). Polyacetylenic thiophenes and terpenoids from the roots of *echinops pappii*. Phytochemistry 30, 879–881. 10.1016/0031-9422(91)85271-Z

[B8] Abegaz B. M. Tadesse M. Majinda R. (1991). Distribution of sesquiterpene lactones and polyacetylenic thiophenes in *Echinops*. Biochem. Syst. Ecol. 19, 323–328. 10.1016/0305-1978(91)90021-Q

[B9] Abera B. (2014). Medicinal plants used in traditional medicine by oromo people, ghimbi district, southwest ethiopia. J. Ethnobiol. Ethnomed. 10, 40. 10.1186/1746-4269-10-40 24885586 PMC4060869

[B10] Abouri M. Mousadik A. E. Msanda F. Boubaker H. Saadi B. Cherifi K. (2012). An ethnobotanical survey of medicinal plants used in the Tata Province, Morocco. Int. J. Med. Plant. Res. 1, 99–123.

[B11] Aburjai T. Darwish R. M. Al-khalil S. Mahafzah A. Al-abbadi A. (2001). Screening of antibiotic resistant inhibitors from local plant materials against two different strains of *Pseudomonas aeruginosa* . J. Ethnopharmacol. 76, 39–44. 11378279 10.1016/s0378-8741(01)00206-9

[B12] Afshaki S. Jafari A. Javidnia K. Firuzi O. (2012). Antioxidant and cytotoxic activities of four plant extracts from dena region of Iran. Res. Pharm. Sci. 7, S853.

[B13] Agrawal M. Nahata A. Dixit V. K. (2012). Protective effects of *echinops echinatus on* testosterone-induced prostatic hyperplasia in rats. Eur. J. Integr. Med. 4, 177–185. 10.1016/j.eujim.2012.01.004

[B14] Ahmad M. Ghafoor N. Aamir M. N. (2012). Antibacterial activity of mother tinctures of cholistan desert plants in Pakistan. Indian. J. Pharm. Sci. 74, 465–468. 10.4103/0250-474X.108429 23716878 PMC3660876

[B15] Akdime H. Boukhira S. Mansouri L. E. L. El A. H. Bousta D. (2015). Ethnobotanical study and traditional knowledge of medicinal plants in ain leuh region (Middle-Atlas of Morocco). Am. J. Adv. Drug. Deliv. 3, 248–263.

[B16] Alam M. K. Ahmed S. Anjum S. Akram M. Shah S. M. Wariss H. M. (2016). Evaluation of antipyretic activity of some medicinal plants from Cholistan desert Pakistan. Pak. J. Pharm. Sci. 29, 529–533. 27087078

[B17] Ameya G. Gure A. Dessalegn E. (2016). Antimicrobial activity of *echinops kebericho* against human pathogenic bacteria and fungi. Afr. J. Tradit. Complement. Altern. Med. 13, 199–203. 10.21010/ajtcam.v13i6.29 PMC5412195 28480380

[B18] Amsalu N. Bezie Y. Fentahun M. Alemayehu A. Amsalu G. (2018). Use and conservation of medicinal plants by indigenous people of gozamin wereda, east gojjam zone of amhara region, ethiopia: an ethnobotanical approach. evidence-based. complement. altern. Med. 2018, 1–23. 10.1155/2018/2973513 PMC5884302 29743921

[B19] Arroo R. R. Jacobs J. J. Van Gestel J. A. Kenkel H. Jannink W. Croes A. F.. (1997). Regulation of thiophene biosynthesis by sulphate in roots of marigolds. New. Phytol. 135, 175–181.

[B20] Asadbeigi M. Mohammadi T. Rafieian-Kopaei M. Saki K. Bahmani M. Delfan M. (2014). Traditional effects of medicinal plants in the treatment of respiratory diseases and disorders: an ethnobotanical study in the Urmia. Asian. Pac. J. Trop. Med. 7, S364–S368. 10.1016/S1995-7645(14)60259-5 25312151

[B21] Asadi M. Hadinedoushan H. Mirghanizadeh S. A. Karimollah A. Dashti F. Malek-hosseini S. (2014). The effect of *echinops lasiolepis* extracts, native plant of yazd province, on peripheral blood mononuclear cellproliferation and IFN-γ Secretion. Int. J. Med. Lab. 1, 7—14.

[B22] Ashebir M. Ashenafi M. (1999). Evaluation of the anibactrtial activity of crude preparation of *zingiber officinale* (zinjibl) *echinops* spp. (kebericho), *coriandrum sativum* (dimbilal), and *cymbopogan citraus* (tej sar) on some food-borne pathogens. Ethiop. J. Hlth. Sci. 9, 33–39.

[B23] Aydın Ç. Özcan G. T. Turan M. Mammadov R. (2016). Phenolic contents and antioxidant properties of *echinops ritro* L. and *E. tournefortii* Jaup. Et. Spach extract. Int. J. Sec. Metabolite. 3, 74–81.

[B24] Baydoun S. Chalak L. Dalleh H. Arnold N. (2015). Ethnopharmacological survey of medicinal plants used in traditional medicine by the communities of mount hermon, Lebanon. J. Ethnopharmacol. 173, 139–156. 10.1016/j.jep.2015.06.052 26165826

[B25] Belay G. Tariku Y. Kebede T. Hymete A. Mekonnen Y. (2011). Ethnopharmacological investigations of essential oils isolated from five ethiopian medicinal plants against eleven pathogenic bacterial strains. Phytopharmacology 1, 133–143.

[B26] Belayneh A. Bussa N. F. (2014). Ethnomedicinal plants used to treat human ailments in the prehistoric place of harla and dengego valleys, eastern ethiopia. J. Ethnobiol. Ethnomed. 1, 18. 10.1186/1746-4269-10-18 PMC3933041 24499509

[B27] Biruksew A. Zeynudin A. Alemu Y. Golassa L. Yohannes M. Debella A. (2018). *Zingiber Officinale* Roscoe and *Echinops Kebericho* Mesfin showed antiplasmodial activities against *Plasmodium berghei* in a dose-dependent manner in Ethiopia. Ethiop. J. Health. Sci. 28, 655. 10.4314/ejhs.v28i5.17 30607081 PMC6308778

[B28] Bitew H. Mammo W. Hymete A. Yeshak M. Y. (2017). Antimalarial activity of acetylenic thiophenes from *echinops hoehnelii* schweinf. Molecules 22, 1965. 10.3390/molecules22111965 PMC6150322 29160791

[B29] Bizuayehu B. Garedew B. (2018). A review on the ethnobotanical study of medicinal plants used for the treatment of gonorrhea disease in Ethiopia. Indian. J. Nat. Prod. Resour. 9, 183–193.

[B30] Bouattour E. Fakhfakh J. Dammak F. Jabou K. Damak M. (2016). Hexane extract of *echinops spinosissimus* turra subsp. spinosus from tunisia: a potential source of acetylated sterols – investigation of its Biological Activities. Chem. Biodivers. 13, 1674–1684. 10.1002/cbdv.2016001182016;1674-84 27476999

[B31] Bouba A. Njintang Y. N. Scher J. Mbofung C. M. F. (2010). Phenolic compounds and radical scavenging potential of twenty cameroonian spices. Agric. Biol. J. N. Am. 1, 213–224.

[B32] Boumaraf M. Benyahia S. Mekkiou R. Benayache S. Benayache F. (2016). Flavonoids from ethyl acetate extract of *echinops spinosus* (Asteraceae). Der Pharma. Chemica. 8, 158–160.

[B33] Bouzabata A. Mahomoodally F. Tuberoso C. (2018). Ethnopharmacognosy of Echinops spinosus L. in North Africa: a mini review. J. Complement. Med. Res. 8, 40–52. 10.5455/jcmr.20180318051853

[B34] Bulut G. Haznedaroğlu M. Z. Doğan A. Koyu H. Tuzlacı E. (2017). An ethnobotanical study of medicinal plants in acipayam (Denizli-Turkey). J. Herb. Med. 10, 64–81. 10.1016/j.hermed.2017.08.001

[B35] Chadwick M. Trewin H. Gawthrop F. Wagstaff C. (2013). Sesquiterpenoids lactones: benefits to plants and people. Int. J. Mol. Sci. 14, 12780–12805. 10.3390/ijms140612780 23783276 PMC3709812

[B36] Chang F. P. Chen C. C. Huang H. C. Wang S. Y. Chen J. J. Yang C. S. (2015). A new bithiophene from the root of *echinops grijsii*. Nat. Prod. Commun. 12, 2147–2149. 26882687

[B37] Chaturvedi M. Mali P. C. Dexit V. P. (1995). Antifertility effects of the roots of *echinops echinatus* (Roxb.) in male rats. J. Phytol. Res. 8, 115–118.

[B38] Chaudhuri P. K. (1987). Echinozolinone, an alkaloid from *echinops echinatus*. Phytochemistry 26, 587–589.

[B39] Chaudhuri P. K. (1992). 7-hydroxyechinozolinone, a new alkaloid from the flowers of *Echinops echinatus*. J. Nat. Prod. 55, 249–250. 10.1021/np50080a019

[B40] Chermat S. Gharzouli R. (2015). Ethnobotanical study of medicinal flora in the north east of algeria - an empirical knowledge in djebel zdimm (Setif). J. Mater. Sci. Eng. A 5, 50–59.

[B41] Dangwal L. R. Rana C. S. Sharma A. (2011). Ethno-medicinal plants from transitional zone of nanda devi biosphere reserve, district chamoli, uttarakhand (India). Indian. J. Nat. Prod. Resour. 2, 116–120.

[B42] Desta B. (1995). Ethiopian traditional herbal drugs. Part I: studies on the toxicity and therapeutic activity of local taenicidal medications. J. Ethnopharmacol. 45, 27–33. 7739224 10.1016/0378-8741(94)01191-2

[B43] Dong L. I. Ning L. I. Wan X. I. Peng Z. H. Zhong-jun M. A. Xian L. I. (2008a). Chemical constituents of the root of *echinops grijisii* Hance. Shenyang. Yao. Ke. Da. Xue. Xue. Bao. 8, 007.

[B44] Dong M. Cong B. Yu S. H. Sauriol F. Huo C. H. Shi Q. W. (2008b). Echinopines A and B: sesquiterpenoids possessing an unprecedented skeleton from *Echinops spinosus.* Org. Lett. 10, 701–704. 10.1021/ol702629z 18251544

[B45] El Abbouyi P. A. Ansari N. F. Khyari P. S. E. Loukili H. (2014). Inventory of medicinal plants prescribed by traditional healers in El Jadida city and suburbs (Morocco). Int. J. Green Pharm. 8, 242–251.

[B46] El-Ghazali G. E. Al-Khalifa K. S. Saleem G. A. Abdallah E. M. (2010). Traditional medicinal plants indigenous to Al-Rass province, Saudi Arabia. J. Med. Plants. Res. 4, 2680–2683. 10.5897/JMPR09.556

[B47] Eram S. Ahmad M. Arshad S. (2013). Experimental evaluation of *echinops echinatus* as an effective hepatoprotective. Sci. Res. Essays. 8, 1919–1923. 10.5897/SRE2012.0766

[B48] Erenler R. Yilmaz S. Aksit H. Sen O. Genc N. Elmastas M. (2014). antioxidant activities of chemical constituents isolated from *echinops orientalis* Trauv. Rec. Nat. Prod. 8, 32–36.

[B49] Etoundi C. B. Kuaté D. Ngondi J. L. Oben J. (2010). Anti-amylase, anti-lipase and antioxidant effects of aqueous extracts of some cameroonian spices. J. Nat. Prod. 3, 165–171.

[B50] Fankam A. G. Kuete V. Voukeng I. K. Kuiate J. R. Pages J. (2011). Antibacterial activities of selected cameroonian spices and their synergistic effects with antibiotics against multidrug-resistant phenotypes. BMC Complement. Altern. Med. 11, 104. 10.1186/1472-6882-11-104 22044718 PMC3228721

[B51] Farouji A. E. Khodayari H. (2016). Ethnomedicinal plants of Farouj district, north khorasan province, Iran. J. Herbal. Drugs. 7, 21–36.

[B52] Fatima S. Afroz S. Qureshi A. S. (2017). Anti-diabetic activity of hydro-alcoholic root extract of *echinops echinatus* and its beneficial effects on nephropathy in experimental rats. Indian. J. Res. Pharm. Biotechnol. 5, 19–27.

[B53] Fenetahun Y. Eshetu G. (2017). A review on ethnobotanical studies of medicinal plants use by agro-pastoral communities in, Ethiopia. J. Med. Plants. Stud. 5, 33–44.

[B54] Fokialakis N. Cantrell C. Duke S. O. Skaltsounis A. L. Wedge D. E. (2006a). Antifungal activity of thiophenes from *echinops ritro*. J. Agric. Food Chem. 54, 1651–1655. 16506815 10.1021/jf052702j

[B55] Fokialakis N. Osbrink W. L. A. Mamonov L. K. Gemejieva N. G. Mims A. B. Skaltsounis A. L. (2006b). Antifeedant and toxicity effects of thiophenes from four *echinops* species against the formosan subterranean termite, *coptotermes formosanus*. Pest. Manag. Sci. 62, 832–838. 10.1002/ps.1237 16791907

[B56] Funk V. A. Bayer R. J. Keeley S. Chan R. Watson L. Gemeinholzer B.., (2005). Everywhere but antarctica: using a super tree to understand the diversity and distribution of the compositae. Biol. Skr. 55, 343–374.

[B57] Gabriel T. Guji T. (2014). Ethnopharmacological survey of medicinal plants in Agaro district, Jimma zone, South West Ethiopia. Int. J. Pharm. Sci. Res. 5, 3551. 10.13040/IJPSR.0975-8232.5(8).3551-59

[B58] Gari A. Yarlagadda R. Wolde-Mariam M. (2015). Knowledge, attitude, practice, and management of traditional medicine among people of Burka Jato Kebele, West Ethiopia. J. Pharm. Bioall. Sci. 7, 136–144. 10.4103/0975-7406.148782 PMC4399012 25883518

[B59] Garnatje T. Susanna A. Garcia-Jacas N. Vilatersana R. Vallès J. (2005). A first approach to the molecular phylogeny of the genus *Echinops* (Asteraceae): Sectional delimitation and relationships with the genus *Acantholepis*. Folia. Geobot. 40, 407–419.

[B60] Ghasemi Pirbalouti A. Momeni M. Bahmani M. (2013). Ethnobotanical study of medicinal plants used by Kurd tribe in Dehloran and Abdanan districts, Ilam province, Iran. Afr. J. Tradit. Complement. Altern. Med. 10, 368–385. 10.4314/ajtcam.v10i2.24 24146463 PMC3746586

[B61] Giday M. Asfaw Z. Woldu Z. (2010). Ethnomedicinal study of plants used by Sheko ethnic group of Ethiopia. J. Ethnopharmacol. 132, 75–85. 10.1016/j.jep.2010.07.046 20674734

[B62] Guo D. A. Lou Z. C. Liu Z. A. (1994). Chemical components of volatile oil from *Echinops grijisii* Hance. Zhongguo. Zhong. Yao. Za. Zhi. 19, 100–101. 8011129

[B63] Gupta R. Vairale M. G. Deshmukh R. R. Chaudhary P. R. Wate S. R. (2010). Ethnomedicinal uses of some plants used by Gond tribe of Bhandara district, Maharashtra. Indian J. Tradit. Knowl. 9, 713–717.

[B64] Hamayun M. Khan M. A. Chudhary M. F. Ahmad H. (2006). Studies on traditional knowledge of medicinal herbs of swat kohistan, District Swat, Pakistan. J. Herbs. Spices. Med. Plants. 12, 11–28. 10.1300/J044v12n04_02

[B65] Hammiche V. Maiza K. (2006). Traditional medicine in central sahara: pharmacopoeia of tassili n'ajjer. J. Ethnopharmacol. 105, 358–367. 10.1016/j.jep.2005.11.028 16414225

[B66] He L. Chao Q. Li R. Lin G. Huang H. (2000). A new pentacyclic triterpene, gmeliniin A, from *Echinops gmelinii* Turcz. Chin. J. Chem. 18, 112–114.

[B67] Hedberg I. Friis I. Edwards S. (2004). “Vol. 4, part 2',” in Flora of Ethiopia and Eritrea (Addis Ababa: Addis Ababa University), 15–23.

[B68] Heshmati S. Madani M. Amjad L. (2016). Study of inhibitory effect of *echinops cephalotes on candida* spp isolated from vulvovaginal candidiasis Patients in Isfahan. J. Res. Med. Sci. 18, e7355. 10.17795/zjrms-7355

[B69] Hussien J. Urgessa K. Regassa F. Jemal A. Abajebel S. Hussien N. (2011). Antihelmentic effects of the essential oil extracts of selected plants against *Haemonchus contortus* . Int. J. Agric. Res. 6, 290–298.

[B70] Hymete A. Kidane A. (1991). Screening for anthelmintic activity in two *Echinops* spp. Ethiop. Pharm. J. 9, 67–71.

[B71] Hymete A. Iversen T. H. Rohloff J. Erko B. (2005a). Screening of *echinops ellenbeckii* and *echinops longisetus* for biological activities and chemical constituents. Phytomedicine 12, 675–679. 10.1016/j.phymed.2004.01.013 16194056

[B72] Hymete A. Rohloff J. Iversen T. H. (2004). Chemical constituents of volatile fractions from *echinops ellenbeckii* O. Hoffm. J. Essent. Oil. Bear Pl. 7, 9–15. 10.1080/0972-060X.2004.10643359

[B73] Hymete A. Rohloff J. Kjosen H. Iversen T. H. (2005b). Acetylenic thiophenes from the roots of *echinops ellenbeckii* from Ethiopia. Nat. Prod. Res. 19, 755–761. 10.1080/1478641012000301711 16317830

[B74] Issa T. O. Mohamed Y. S. Yagi S. Ahmed R. H. Najeeb T. M. Makhawi A. M. (2018). Ethnobotanical investigation on medicinal plants in Algoz area (South Kordofan), Sudan. J. Ethnobiol. Ethnomed. 14, 1–22. 10.1186/s13002-018-0230-y 29699576 PMC5921783

[B75] Jabeen N. Ajaib M. Siddiqui M. F. Ulfat M. Khan B. (2015). A survey of ethnobotanically important plants of District Ghizer, Gilgit-Baltistan. FUUAST. J. Biol. 5, 153–160.

[B76] Jiang B. Wang F. Liu L. Tian S. Li W. Yang X. (2017). Antibacterial activity and action mechanism of the *echinops ritro* L. essential oil against foodborne pathogenic bacteria. J. Essent. Oil Bear. Pl. 20, 1172–1183. 10.1080/0972060X.2017.1399090

[B77] Jin Q. Lee J. W. Jang H. Choi J. E. Kim H. S. Lee D. (2016). Dimeric sesquiterpene and thiophenes from the roots of *Echinops latifolius.* Bioorg. Med. Chem. Lett. 26, 5995–5998. 10.1016/j.bmcl.2016.10.017 27865705

[B78] Jin W. Sh i Q. Hong C. Cheng Y. Ma Z. Qu H. (2008). Cytotoxic properties of thiophenes from *echinops grijissi* Hance. Phytomedicine 15, 768–774. 10.1016/j.phymed.2007.10.007 18068965

[B79] Ka S. (2001). A pseudoguaiane sesquiterpene xylopyranoside *from Echinops hussoni*. Pharmazie 56, 415–417. 11400560

[B80] Kamatenesi M. M. Acipa A. Oryem-Origa H. (2011). Net medicinal plants of otwal and ngai sub counties in oyam district, northern uganda. J. Ethnobiol. Ethnomed. 7, 1–14. 10.1186/1746-4269-7-7 21241484 PMC3029220

[B81] Kamte S. L. N. Ranjbarian F. Campagnaro G. D. Nya P. C. B. Woguem V. Womeni H. M. (2017). *Trypanosoma brucei* Iinhibition by essential oils from medicinal and aromatic plants traditionally used in cameroon (*Azadirachta indica*, *Aframomum melegueta*, *Aframomum daniellii*, *Clausena anisata*, *Dichrostachys cinerea* and *Echinops giganteus*). Int. J. Environ. Res. Public. Health. 14, 737. 10.3390/ijerph14070737 PMC5551175 28684709

[B82] Karimov U. T. Aisa H. A. (2012). Phytochemical study of the plant Echinops integrifolius growing in the Altai (XUAR PRC). Chem.Nat. Compd. 48, 903–905

[B83] Karimov U. T. Aisa H. A. (2013). Hydrocarbons and fatty acids from *echinops integrifolius*. Chem. Nat. Compd 49, 920–921. 10.1007/s10600-013-0778-7

[B84] Karunamoorthi K. Mulelam A. Wassie F. (2008). Laboratory evaluation of traditional insect/mosquito repellent plants against anopheles arabiensis, the predominant malaria vector in ethiopia. Parasitol. Res. 103, 529–534. 10.1007/s00436-008-1001-9 18493796

[B85] Katiri A. Barkaoui M. Msanda F. Boubaker H. (2017). Ethnobotanical survey of medicinal plants used for the treatment of diabetes in the tizi n' test region (Taroudant Province, Morocco). J. Pharmacogn. Nat. Prod. 03, 1–10. 10.4172/2472-0992.1000130

[B86] Kevin K. John K. Carolyn N. Derrick S. Lubega A. (2018). *In* vitro anti-tuberculosis activity of total crude extract of *echinops amplexicaulis* against multi-drug resistant *Mycobacterium tuberculosis*. J. Health. Sci 6, 296–303. 10.17265/2328-7136/2018.04.008

[B87] Khadim E. J. Abdulrasool A. A. Awad Z. J. (2014). Phytochemical investigation of alkaloids in the iraqi *echinops heterophyllus* (Compositae). Iraqi J. Pharm. Sci. 23, 26–34.

[B88] Kitata G. Abdeta D. Amante M. (2017). Ethnoknowledge of plants used in veterinary practices in midakegn district, west showa of oromia region, Ethiopia. J. Med. Plants. Stud. 5, 282–288.

[B89] Kiyekbayeva L. Mohamed N. M. Yerkebulan O. Mohamed E. I. Ubaidilla D. Nursulu A. (2017). Phytochemical constituents and antioxidant activity of *echinops albicaulis*. Nat. Prod. Res. 32, 1203–1207. 10.1080/14786419.2017.1323213 28475371

[B90] Kuete V. Krusche B. Youns M. Voukeng I. Fankam A. G. Tankeo S. (2011). Cytotoxicity of some cameroonian spices and selected medicinal plant extracts. J. Ethnopharmacol. 134, 803–812. 10.1016/j.jep.2011.01.035 21291988

[B91] Kuete V. Sandjo L. P. Wiench B. Efferth T. (2013). Cytotoxicity and modes of action of four cameroonian dietary spices ethno-medically used to treat cancers: *echinops giganteus*, *xylopia aethiopica*, *imperata cylindrica* and *piper capense*. J. Ethnopharmacol. 149, 245–253. 10.1016/j.jep.2013.06.029 23827757

[B92] Kumar H. Khajuria A. K. Bisht N. S. (2018). Traditional phytoremedies used to treatment urolithiasis in Pauri (Garhwal) Uttarakhand India. J. Pharmacogn. Phytochem. 7, 2941–2944.

[B93] Kumar S. Pandey S. (2015). An ethnobotanical study of local plants and their medicinal importance in tons river area, dehradun, uttarakhand. Indian. J. Trop. Biodivers. 23, 227–231.

[B94] Lamorde M. Tabuti J. R. S. Obua C. Kukunda-Byobona C. Lanyero H. Byakika-Kibwika P. (2010). Medicinal plants used by traditional medicine practitioners for the treatment of HIV/AIDS and related conditions in Uganda. J. Ethnopharmacol. 130, 43–53. 10.1016/j.jep.2010.04.004 20451595

[B95] Li L. Ren J. Cheng Z. Zhu H. (2010). Three new sesquiterpenoids from *echinops ritro* L. Helve. Chimica. Acta. 93, 1344–1349.

[B96] Li L. B. Xiao G. D. Xiang W. Yang X. Cao K. X. Huang R. S. (2019). Novel substituted thiophenes and sulf-polyacetylene ester from *Echinops ritro* L. Molecules 24, 805. 10.3390/molecules24040805 PMC6413031 30813374

[B97] Lin C. C. Lin C. H. (1993). Pharmacological and pathological studies on taiwan folk medicine (IX): the heptoprotective effect of the methanolic extract of *Echinops grijissi*. Am. J. Chin. Med. XXI, 21, 33–34. 10.1142/S0192415X93000054 8328420

[B98] Lin C. C. Lin C. H. Chiu H. F. Hu M. F. (1992). Pharmacological and pathological studies on taiwan folk medicine (VII): the anti-inflammatory effect of *Echinops grijissi* . Am. J. Chin. Med. XX, 20, 127–134. 10.1142/S0192415X92000138 1519553

[B99] Liu Y. Ye M. Guo H. Z. Zhao Y. Y. Guo D. A. (2002). New thiophenes from *echinops grijisii*. J. Asian. Nat. Prod. Res. 4, 175–178. 10.1080/1028602021000000071 12118504

[B100] Mahmood A. A. R. Khadeem E. J. (2013). Phytochemical investigation of flavonoids glycoside in the Iraqi *Echinops heterophyllus* (Compositae). Pharm. Glob. 4, 1.

[B101] Mahmoud T. Gairola S. (2013). Journal of medicinal plants studies traditional knowledge and use of medicinal plants in the eastern desert of egypt : a case study from Wadi El-Gemal National Park. J. Med. Plants. Stud. 1, 10–17.

[B102] Malik K. Ahmad M. Zhang G. Rashid N. Zafar M. Sultana S. (2018). Traditional plant based medicines used to treat musculoskeletal disorders in Northern Pakistan. Eur. J. Integr. Med. 19, 17–64. 10.1016/j.eujim.2018.02.003

[B103] Maru R. N. Patel H. R. Patel R. S. (2018). Some plants used for the treatment of eye and earche from forest areas of Jhalod Taluka, Dahod District, Gujarat, India. Int. J. S. Res. Sci. Tech. 4, 578–581.

[B104] Maurya S. K. Kushwaha A. K. Seth A. (2015). Ethnomedicinal review of Usnakantaka (*Echinops echinatus* Roxb.). Pharmacogn. Rev. 9, 149–154. 10.4103/0973-7847.162138 26392713 PMC4557238

[B105] Mekuanent T. Zebene A. Solomon Z. (2015). Ethnobotanical study of medicinal plants in chilga district, northwestern ethiopia. J. Nat. Remedies. 15, 88–112. 10.18311/jnr/2015/476

[B106] Menut C. Lamaty G. Weyerstahl P. Marschall H. Seelmann I. Amvam Zollo P. H. (1997). Aromatic plants of tropical Central Africa. Part XXXI. Tricyclic sesquiterpenes from the root essential oil of Echinops giganteus var. lelyi CD Adams. Flavour. Frag. J. 12, 415–421

[B107] Meragiaw M. Asfaw Z. Argaw M. (2016). The status of ethnobotanical knowledge of medicinal plants and the impacts of resettlement in Delanta, northwestern Wello, northern Ethiopia. Evid. Based. Complement. Alternat. Med. 2016, 24. 10.1155/2016/5060247 PMC4737471 26881004

[B108] Merzouki A. Ed-derfoufi F. Mesa J. M. (2000). Contribution to the knowledge of Rifian traditional medicine. II: folk medicine in Ksar Lakbir district (NW Morocco). Fitoterapia. 71, 278–307. 10844168 10.1016/s0367-326x(00)00139-8

[B109] Mesfin F. Seta T. Assefa A. (2014). An ethnobotanical study of medicinal plants in Amaro Woreda Ethiopia. Ethnobot. Res. Appl. 12, 341–354.

[B110] Miao C. G. Zhou G. L. Qin M. S. Chen J. Z. Li C. F. Zhang B. (2015). Treatment of rheumatoid arthritis with flavonoids of *echinps latifolius* tausch in rat model. Zhejiang. Da. Xue. Xue. Bao. Yi. Xue. Ban. 44, 43–48. 25851974 10.3785/j.issn.1008-9292.2015.1.007 PMC10397049

[B111] Mohseni S. Sani A. M. Tavakoli M. Raoufi A. M. (2017). Effect of extraction conditions on antioxidant activities of *echinops persicus*. J. Essent. Oil. Bear. Pl. 20, 1633–1644. 10.1080/0972060X.2017.1399088

[B112] Moravec I. Fernández E. Vlkova M. Milella L. (2014). Ethnobotany of medicinal plants of northern ethiopia. Bol. Latinoam. Caribe. Plantas. Me. Aromát. 13, 126–134.

[B113] Mustafa B. Hajdari A. Krasniqi F. Hoxha E. Ademi H. Quave C. L. (2012). Medical ethnobotany of the albanian alps in kosovo. J. Ethnobiol. Ethnomed. 8, 1–14. 10.1186/1746-4269-8-6 22284581 PMC3285519

[B114] Nahata A. Dixit V. K. (2014). Evaluation of 5α-reductase inhibitory activity of certain herbs useful as antiandrogens. Andrologia. 46, 592–601. 10.1111/and.12115 23710567

[B115] Nakano H. Ali A. Rehman J. U. Mamonov L. K. Cantrell C. L. Khan I. A. (2014). Toxicity of thiophenes from *echinops transiliensis* (Asteraceae) against *a aegypti* (Diptera: Culicidae) larvae. Chem. Biodivers. 11, 1001–1009. 25044586 10.1002/cbdv.201300290

[B116] Nakano H. Cantrell C. L. Mamono L. K. Osbrink W. L. A. Ross S. A. (2011). Echinopsacetylenes A and B, new thiophenes from *Echinops transiliensis*. Org. Lett. 13, 6228–6231. 10.1021/ol202680a 22066834

[B117] Nakano H. Cantrell C. L. Mamonov L. K. Kustova T. S. Fronczek F. R. Ross S. A. (2012). Chemical constituents from *Echinops nanus* and *Echinops transiliensis*. Biochem. Syst. Ecol. 45, 127–129. 10.1016/j.bse.2012.07.008

[B118] Nawash O. Shudiefat M. Al-Tabini R. Al-Khalidi K. (2013). Ethnobotanical study of medicinal plants commonly used by local bedouins in the badia region of Jordan. J. Ethnopharmacol. 148, 921–925. 10.1016/j.jep.2013.05.044 23727184

[B119] Nyang'au H. O. Maingi J. Kebira A. Muriithi I. A. Muthoni N. P. Ntarara M. G. (2017). The efficacy of some medicinal plants used locally within Transmara west, Narok County, Kenya against selected *Enterobacteria* and *Candida*. Infection 11, 13.

[B120] Okello J. Ssegawa P. (2007). Medicinal plants used by communities of Ngai Subcounty, Apac District, northern Ugand. Afr. J. Ecol. 45, 76–83.

[B121] Padashetty S. A. Mishra S. H. (2007). Effect of terpenoidal fraction of *Echinops echinatus* roots on reproductive parameters of male rats. J. Nat. Med. 61, 452–457. 10.1007/s11418-007-0173-4

[B122] Papadopoulou P. Couladis M. Tzakou O. (2006). Essential Oil Composition of two Greek *Echinops* species: *E. graecus* Miller and *E. ritro* L. J. Essent. Oil. Res. 18, 242–243. 10.1080/10412905.2006.9699076

[B123] Patel A. J. (2016). Isolation and characterization of lupeol from *Echinops echinatus* Roxb. Root. Eur. J. Pharm. Med. Res. 3, 385–387.

[B124] Patel A. J. Patel N. M. Patel A. A. Patel J. Patel S. (2011b). Comparative analgesic activity of root and aerial part methanolic extracts of *Echinops echinatus* Roxb. Int. J. Pharm. Innov. 1, 23–29.

[B125] Patel A. J. Patel N. M. Patel A. A. Patel J. Patel S. (2011a). Comparative diuretic activity of root and aerial part methanolic extracts of *Echinops echinatus* Roxb. Pharm. Lett. 3, 168–172.

[B126] Pavela R. Maggi F. Mbuntcha H. Woguem V. Fogang H. P. D. Womeni H. M. (2016). Traditional herbal remedies and dietary spices from cameroon as novel sources of larvicides against filariasis mosquitoes? Parasitol. Res. 115, 4617–4626. 10.1007/s00436-016-5254-4 27679452

[B127] Qureshi R. Bhatti G. R. (2008). Ethnobotany of plants used by the Thari people of Nara Desert, Pakistan. Fitoterapia. 79, 468–473. 10.1016/j.fitote.2008.03.010 18538950

[B128] Rad A. A. Najafzadeh-Varzi H. Farajzadeh-Sheikh A. (2010). Evaluation of anti-ulcer activity of *echinops persicus* on experimental gastric ulcer models in rats. Vet. Res. Forum. 1, 188–189.

[B129] Radulović N. S. Denić M. S. (2013). Essential oils from the roots of *echinops bannaticus* rochel ex schrad. and *echinops sphaerocephalus* L.(Asteraceae): chemotaxonomic and biosynthetic aspects. Chem. Biodivers. 10, 658–676. 10.1002/cbdv.201200330 23576352

[B130] Rahman S. M. Abd-Ellatif S. A. Deraz A. F. Khalil A. A. (2011). Antibacterial activity of some wild medicinal plants collected from western mediterranean coast, Egypt: natural alternatives for infectious disease treatment. Afr. J. Biotechnol. 10, 10733–10743. 10.5897.AJB11.007

[B131] Ram S. N. Roy R. Singh B. Singh R. P. Pandey V. B. (1995). An acylfiavone glucoside of *echinops echinatus* flowers. Planta. Med. 62, 187. 10.1055/s-2006-957855 17252438

[B132] Rathore S. Tiwari J. K. Malik Z. A. (2015). Ethnomedicinal survey of herbaceous flora traditionally used in health care practices by inhabitants of dhundsir gad watershed of garhwal himalaya, India. Global. J. Res. Med. Plants. Indigen. Med. 4, 65–78.

[B133] Regassa R. (2013). Assessment of indigenous knowledge of medicinal plant practice and mode of service delivery in Hawassa city, southern Ethiopia. J. Med. Plants. 7, 517–535.

[B134] Regassa R. Bekele T. Megersa M. (2017). Ethnobotonical study of traditional medicinal plants used to treat human ailments by halaba people, southern Ethiopia. J. Med. Plants. Stud. 36, 36–47.

[B135] Rimbau V. Cerdan C. Vila R. Iglesias J. (1999). Antiinflammatory activity of some extracts from plants used in the traditional medicine of North-African countries (II). Phytother. Res. 13, 128–132. 10190185 10.1002/(SICI)1099-1573(199903)13:2<128::AID-PTR399>3.0.CO;2-7

[B136] Sajjad A. Adnan S. Hasnain Alam H. Mohamed E. A. R. (2017). Ethno botanical study of traditional native plants in Al Ain UAE. Int. J. Adv. Res. Biol. Sci. 4, 165–174. 10.22192/ijarbs.2017.04.02.020

[B137] Saleh Fares G. O. Abdallah L. Almasri M. Slaileh A. Zurba Z. (2013). Antibacterial activity of selected palestinian wild plant extracts against multidrug-resistant clinical isolate of *streptococcus pneumoniae* . JPR : BioMedRx: Int J. 1, 963–969.

[B138] Sánchez-Jiménez I. Lazkov G. A. Hidalgo O. Garnatje T. (2010). Molecular systematics of *Echinops* L. (Asteraceae, Cynareae): A phylogeny based on ITS and trnL-trnF sequences with emphasis on sectional delimitation. Taxon. 59, 698–708.

[B139] Sandjo L. P. Kuete V. Siwe X. N. Poumale H. M. P. (2016). Cytotoxicity of an unprecedented brominated oleanolide and a new furoceramide from the Cameroonian spice, *Echinops giganteus* . Nat. Prod. Res. 30, 2529–2537. 10.1080/14786419.2015.1120724 26733224

[B140] Sarvaiya D. D. Sheth N. R. Dudhrejiya A. V. (2017). Antidiabetic and antioxidant activity of roots of *Echinops echinatus* Roxb. Pharmacologyonline. 2, 10–39.

[B141] Sathiyamoorthy P. Lugasi-Evgi H. Schlesinger P. Kedar I. Gopas J. Pollack Y. (1999). Screening for cytotoxic and antimalarial activities in desert plants of the negev and bedouin market plant products. Pharm. Biol. 37, 188–195.

[B142] Senejoux F. Demougeot C. Karimov U. Muyard F. Kerram P. Aisa H. A. (2013). Chemical constituents from *echinops integrifolius*. Biochem. Syst. Ecol. 47, 42–44. 10.1016/j.bse.2012.10.013

[B143] Sharma J. Gairola S. Gaur R. D. Painuli R. M. (2012). The treatment of jaundice with medicinal plants in indigenous communities of the sub-himalayan region of uttarakhand, India. J. Ethnopharmacol. 143, 262–291. 10.1016/j.jep.2012.06.034 22759701

[B144] Sharma P. K. Chauhan N. S. Lal B. (2004). Observations on the traditional phytotherapy among the inhabitants of Parvati valley in western Himalaya, India. J. Ethnopharmacol. 92, 167–176. 10.1016/j.jep.2003.12.018 15137998

[B145] Shende A. N. Mohture V. M. Korpenwar A. N. (2018). Traditional medicinal plants used against various diseases in Nagbhid tahsil, Chandrapur (MS) India. Int. J. Life. Sci. A 12, 135–142.

[B146] Shi J. Zhang X. Jiang H. (2010). 2-(Penta-1,3-diynyl)-5-(3,4-dihydroxybut-1-ynyl)thiophene, a novel NQO1 inducing agent from *Echinops grijsii* Hance. Molecules 15, 5273–5281. 10.3390/molecules15085273 20714298 PMC6257798

[B147] Shilema A. Zerom K. Mussa A. (2013). Ethnoveterinary practices against animal trypanosomosis in Amaro district, Southern Ethiopia. Int. J. Med. Plants Res. 2, 238–241.

[B148] Sing B. Ram S. N. Pandeys V. B. Joshis V. K. Gambhir S. S. (1991). Studies on antiinflammatory activity of taraxasterol acetate from *echinops echinatus* in rats and mice. Phytother. Res. 5, 103–106.

[B149] Singh B. Gambhir S. S. Pandjwb V. B. Joshf V. K. (1989). Anti-inflammatory activity of *Echinops echinatus*. J. Ethnopharmacol. 25, 189–199. 2747253 10.1016/0378-8741(89)90021-4

[B150] Singh R. P. Pandey V. B. (1990). Nivetin, a neoflavonoid from *Echinops niveus.* Phytochemistry. Phytochemistry 29, 680–681.

[B151] Singh R. P. Singh K. N. Pandey V. B. (1990). Constituents of *echinops niveus* . Fitoterapia. 61, 279.

[B152] Singh S. Upadhyay R. K. Pandey M. B. Singh J. P. Pandey V. B. (2006). Flavonoids from Echinops echinatus. J Asian Nat Prod Res. 8, 197–200. 16864424 10.1080/1028602042000324826

[B153] Su Y. F. Luo Y. Guo C. Y. Guo D. A. (2004). Two new quinoline glycoalkaloids from *Echinops gmelinii*. J. Asian. Nat. Prod. Res. 6, 223–227. 10.1080/10286020310001653327 15224421

[B154] Suleman S. Alemu T. (2012). A survey on utilization of ethnomedicinal plants in Nekemte town, East Wellega (Oromia), Ethiopia. J. Herbs. Spices. Med. Plants. 18, 34–57. 10.1080/10496475.2011.645188

[B155] Tacham W. N. Fonge B. A. Fonkou T. (2015). Traditional medicine and ethnobotanical use of wild plants by the Mundani people of Wabane, South West Region, Cameroon. J. Ethnobiol. Tradit. Med. Photon. 125, 1060–1080.

[B156] Tariku Y. Hymete A. Hailu A. Rohloff J. (2011). *In vitro* evaluation of antileishmanial activity and toxicity of essential oils of *artemisia absinthium* and *echinops kebericho*. Chem. Biodivers. 8, 614–623. 21480507 10.1002/cbdv.201000331

[B157] Tbatou M. A. Belahyan A. B. Belahsen R. E. (2016). Wild edible plants traditionally used in the countryside of El Jadida, coastal area in the Center of Morocco. Life. Sci. Leafl. 75, 28–48.

[B158] Teklay A. Abera B. Giday M. (2013). An ethnobotanical study of medicinal plants used in Kilte Awulaelo District, Tigray Region of Ethiopia. J. Ethnobiol. Ethnomed. 9, 65. 10.1186/1746-4269-9-65 24011232 PMC3852788

[B159] Tekle Y. (2014). An ethno-veterinary botanical survey of medicinal plants in Kochore district of Gedeo Zone, Southern Nations Nationalities and Peoples Regional State (SNNPRs), Ethiopia. J. Sci. Innov. Res. 3, 433–445.

[B160] Tekwu E. M. Askun T. Kuete V. Nkengfack A. F. Nyasse B. Etoa F. X. (2012). Antibacterial activity of selected cameroonian dietary spices ethno-medically used against strains of *Mycobacterium tuberculosis*. J. ethnopharmacol. 142, 374–382. 10.1016/j.jep.2012.05.003 22595661

[B161] Tene M. Tane P. Sondengam B. L. Connolly J. D. (2004). Lignans from the roots of *echinops giganteus*. phytochemistry 65, 2101–2105. 10.1016/j.phytochem.2004.05.014 15279979

[B162] Tiwari J. K. Ballabha R. Tiwari P. (2010). Ethnopaediatrics in garhwal Himalaya, Uttarakhand, India (Psychomedicine and Medicine). N. Y. Sci. J. 3, 123–126.

[B163] Toma A. Deyno S. Fikru A. Eyado A. Beale A. (2015). *In vivo* antiplasmodial and toxicological effect of crude ethanol extract of *Echinops kebericho* traditionally used in treatment of malaria in Ethiopia. Malar. J. 14, 196. 10.1186/s12936-015-0716-1 25958112 PMC4429705

[B164] Toroğlu S. Keskin D. Vural C. Kertmen M. Cenet M. (2012). Comparison of antimicrobial activity of *echinops viscosus* subsp. *bithynicus* and *E. microcephalus* leaves and flowers extracts from turkey. Int. J. Agric. Biol. 14, 637–640.

[B165] Wagh V. V. Jain A. K. (2018). Status of ethnobotanical invasive plants in western Madhya Pradesh, India. S. Afr. J. Bot. 114, 171–180. 10.1016/j.sajb.2017.11.008

[B166] Wang Y. Li X. Meng D. Li N. Zhang Y. (2008). Chemical constituents of thiophenes from *Echinops latifolius* Tausch. Shenyang. Yao. Ke. Da. Xue. Xue. Bao. 8.

[B167] Wang Y. Li X. Li L. Meng D. Li Z. Li N. (2007). Two new thiophenes from *Echinops latifolius* and their phototoxic activities. Planta. Med. 73, 696–698. 10.1055/s-2007-981541 17564953

[B168] Wang Y. Li X. Meng D.-L. Li Z.-L. Zhang P. Xu J. (2006). Thiophenes from *Echinops latifolius*. Nat. Prod. Res. 8, 585–588. 10.1080/10286020500176724 17135040

[B169] Wang M. Sun J. Jiang Z. Xie W. Zhang X. (2015). Hepatoprotective effect of kaempferol against alcoholic liver injury in mice. Am. J. Chin. Med. 43, 241–254. 25787296 10.1142/S0192415X15500160

[B170] Xu D. G. Lv W. Dai C. Y. Zhu F. F. Xu G. H. Ma Z. J. (2015). 2 2-(Pro-1-ynyl)-5-(5,6-dihydroxypenta-1,3- diynyl) thiophene induces apoptosis through reactive oxygen species-mediated JNK activation in human colon Cancer SW620 Cells. Anat. Rec. 298, 376–385. 10.1002/ar.23045 25178491

[B171] Yadava R. N. Singh S. K. (2006). New anti-inflammatory active flavanone glycoside from the *Echinops echiantus* Roxb. Indian. J. Chem. 45, 1004–1008.

[B172] Yigezu Y. Haile D. B. Ayen W. Y. (2014). Ethnoveterinary medicines in four districts of jimma zone, Ethiopia: cross sectional survey for plant species and mode of use. BMC. Vet. Res. 10, 1–12. 10.1186/1746-6148-10-76 24679045 PMC3978085

[B173] Zamzami T. A. Abdallah H. M. Shehata I. A. Mohamed G. A. Alfaifi M. Y. Elbehairi S. E. I. (2019). Macrochaetosides A and B, new rare sesquiterpene glycosides from *Echinops macrochaetus* and their cytotoxic activity. Phytochem. Lett. 30, 88–92. 10.1016/j.phytol.2019.01.025

[B174] Zhang P. Jin W. R. Shi Q. He H. Ma Z. J. (2008). Two novel thiophenes from *Echinops grijissi* Hance. J. Asian. Nat. Prod. Res. 10, 977–981. 10.1080/10286020802240467 19003618

[B175] Zhang P. Liang D. Jin W. Qu H. Cheng Y. L, i X. (2009). Cytotoxic thiophenes from the root of *Echinops grijisii* Hance. Zeitschrift für. Naturforschung C. 1, 193–196. 10.1515/znc-2009-3-407 19526711

[B176] Zhang X. Ma Z. (2010). Characterization of bioactive thiophenes from the dichloromethane extract of *Echinops grijisii* as Michael addition acceptors. Anal. Bioanal. Chem. 39, 1975–1984. 10.1007/s00216-010-3729-1 20437033

[B177] Zhang H. Tan X. Yang D. Lu J. Liu B. Baiyun (2017). Dietary luteolin attenuates chronic liver injury induced by mercuric chloride via the Nrf2/NF-κB/P53 signaling pathway in rats. Oncotarget. 8, 40982. 10.18632/oncotarget.17334 28498799 PMC5522226

[B178] Zhao M. P. Liu Q. Z. Liu Q. Liu Z. L. (2017). Identification of Larvicidal Constituents of the Essential Oil of *Echinops grijsii* Roots against the three species of mosquitoe. Molecules 22, 205. 10.3390/molecules2202020

[B179] Zhu R. Wang Y. Zhang L. Guo Q. (2012). Oxidative stress and liver disease. Hepatol. Res. 42, 741–749. 10.1111/j.1872-034X.2012.00996.x 22489668

